# Controlling condensation and frost growth with chemical micropatterns

**DOI:** 10.1038/srep19131

**Published:** 2016-01-22

**Authors:** Jonathan B. Boreyko, Ryan R. Hansen, Kevin R. Murphy, Saurabh Nath, Scott T. Retterer, C. Patrick Collier

**Affiliations:** 1 Department of Biomedical Engineering and Mechanics, Virginia Tech, Blacksburg, Virginia 24061, USA; 2 Center for Nanophase Materials Sciences, Oak Ridge National Laboratory, Oak Ridge, Tennessee 37831, USA; 3 Bredesen Center for Interdisciplinary Research and Graduate Education, The University of Tennessee, Knoxville, Tennessee 37996, USA; 4 Chemical Engineering Department, Kansas State University, Manhattan, Kansas 66506, USA; 5 Biosciences Division, Oak Ridge National Laboratory, Oak Ridge, Tennessee 37831, USA

## Abstract

In-plane frost growth on chilled hydrophobic surfaces is an inter-droplet phenomenon, where frozen droplets harvest water from neighboring supercooled liquid droplets to grow ice bridges that propagate across the surface in a chain reaction. To date, no surface has been able to passively prevent the in-plane growth of ice bridges across the population of supercooled condensate. Here, we demonstrate that when the separation between adjacent nucleation sites for supercooled condensate is properly controlled with chemical micropatterns prior to freezing, inter-droplet ice bridging can be slowed and even halted entirely. Since the edge-to-edge separation between adjacent supercooled droplets decreases with growth time, deliberately triggering an early freezing event to minimize the size of nascent condensation was also necessary. These findings reveal that inter-droplet frost growth can be passively suppressed by designing surfaces to spatially control nucleation sites and by temporally controlling the onset of freezing events.

Frost inevitably forms on any subfreezing surface whose temperature is beneath the dew point. Water vapor can bypass the liquid phase and transform directly into ice, known as deposition or desublimation. However, in many cases the water vapor first condenses into supercooled liquid, which later freezes into ice. This indirect method of frost formation is known as condensation frosting and is more common than direct deposition in many cases, particularly at low supersaturation degrees and/or on hydrophobic surfaces^1^.

Until very recently, it was widely assumed that the primary mechanism of condensation frosting for surfaces promoting dropwise condensation was the heterogeneous nucleation of ice at the solid-liquid interface of each individual supercooled droplet^2^,^3^,^4^,^5^. Numerous reports have observed that heterogeneous nucleation can be delayed by minutes or even hours for supercooled droplets deposited on superhydrophobic surfaces compared to traditional surfaces, due to the hydrophobicity increasing the energy barrier for nucleation^6^,^7^,^8^ and because the air pockets trapped by a suspended Cassie droplet reduce the heat transfer rate^8^,^9^,^10^ and minimize the solid-liquid contact area where nucleation events can occur^6^,^7^,^8^,^10^,^11^,^12^. Condensed droplets can also grow in a Cassie state on nanostructured superhydrophobic surfaces^13^,^14^,^15^, such that supercooled condensate can exhibit delayed heterogeneous nucleation in the same manner as with deposited droplets^3^,^4^,^5^. Furthermore, the minimal contact angle hysteresis of Cassie droplets on superhydrophobic surfaces can be exploited to dynamically remove supercooled droplets before heterogeneous nucleation occurs at all. Examples include deposited droplets that were gravitationally removed by sliding^9^,^11^ or rebound^16^,^17^,^18^ and condensed droplets that were removed by gravity at millimetric length scales^19^,^20^ or by coalescence-induced jumping at micrometric length scales^21^,^22^,^23^. It would therefore seem that nanostructured superhydrophobic surfaces should be able to completely prevent the onset of condensation frosting, by delaying heterogeneous nucleation long enough to dynamically remove supercooled condensate from the surface.

Curiously, it was observed that even when supercooled condensate was continually removed from a superhydrophobic surface via coalescence-induced jumping before heterogeneous nucleation occurred, frost still grew over the surface^21^. This was attributed to an unexpected phenomenon where only a single droplet had to freeze due to heterogeneous nucleation (typically at edge defects where jumping did not occur), at which point the frozen droplet harvested water from nearby liquid droplets, growing ice bridges that connected across the forming condensate in an unstoppable chain reaction^21^,^24^. This source-sink vapor interaction between an evaporating liquid droplet and a bridging frozen droplet is most likely due to the higher water vapor pressure over liquid water compared to frozen water^25^,^26^,^27^. Since the mass of the ice bridge is harvested from the liquid droplet, it follows that the liquid droplet can completely evaporate before the ice bridge connects when the inter-droplet spacing is sufficiently large^21^,^24^. To date, no surface has been able to completely stop the inter-droplet growth of frost when a freezing event occurs near the population of supercooled condensate^24^,^28^,^29^,^30^,^31^; typically ice bridges connect for nearly every droplet on smooth hydrophobic surfaces and for approximately 1/3 of droplets on jumping-droplet superhydrophobic surfaces^21^.

Inspired by the *Stenocara* desert beetle^32^, the nucleation sites for condensation can be spatially controlled on engineered surfaces exhibiting chemical patterns of contrasting hydrophobic and hydrophilic regions, due to the dramatically lower energy barrier for nucleation on the hydrophilic features^33^,^34^,^35^,^36^,^37^,^38^. Chemically patterned substrates have been shown to enhance water harvesting^39^,^40^,^41^ and phase-change heat transfer^42^,^43^,^44^, and are also useful for controlling liquid morphology^45^,^46^, tuning droplet hysteresis^47^,^48^, controllably depositing micro/nano-materials^49^, and lab-on-a-chip applications^50^,^51^. Now that it is understood that condensation frosting is dependent upon the spatial distribution of droplets, the ability of a chemically patterned surface to control nucleation sites seems ideal for characterizing and controlling inter-droplet frost growth. However, to date there have been no reports using chemically patterned surfaces to characterize the spatial distribution of condensation or the related dynamics of inter-droplet frost growth.

Here, we demonstrate that chemical patterns can be used to tune the spatial distribution of supercooled condensation and subsequently control the geometry and speed of inter-droplet frost growth. The success and rate of inter-droplet frost growth was found to be dependent upon two primary factors: the extent of spacing between hydrophilic regions where liquid nucleation occurred and the time allowed for condensation growth prior to the initial freezing event. For the first time, inter-droplet ice bridging could be completely halted by utilizing sufficiently sparse hydrophilic patterns and by quickly triggering a freezing event near the patterned condensation.

## Results

### Chemical micropatterns

**Figure 1:**
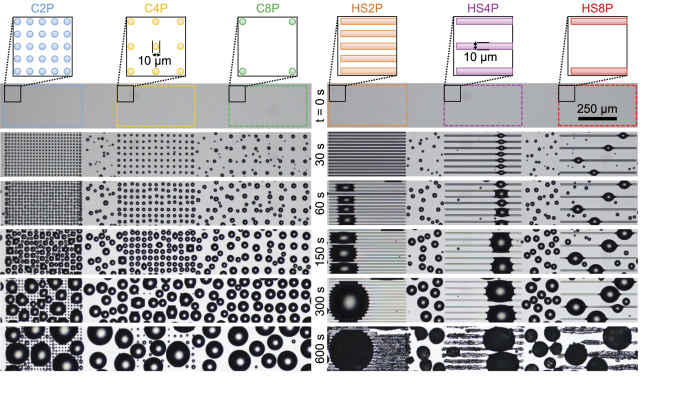
Spatial control of condensation on smooth chemical micropatterns composed of arrays of circles or stripes. The colored shapes in the schematics represent the hydrophilic features while the white background is hydrophobic. Time zero corresponds to the onset of cooling from 10 °C down to a steady-state temperature of *T*_*s*_ = −10 °C from 30 s onward. See Supplementary Movies 2 and 4.

Arrays of hydrophilic features were patterned against a hydrophobic backdrop on a silicon wafer using photolithography (see Methods and Supplementary Fig. S1). The hydrophilic areas were composed of bare silicon oxide and the hydrophobic areas were comprised of a silane monolayer. Immediately before experimental characterization, each wafer was exposed to an oxygen plasma to restore the full hydrophilicity of the silicon oxide features, followed by a dry peel-off of the patterned parylene coating^52^ that was protecting the hydrophobic monolayer. Atomic force microscopy revealed that the silanized regions were uniformly elevated by 1.5 ± 0.5 nm relative to the bare hydrophilic features, confirming the presence of a self-assembled monolayer (Supplementary Fig. S2). This extreme smoothness is ideal for characterizing the effects of wettability patterns on condensation and frost growth without any conflating topological effects such as polymers^36^,^40^, surface deformation from a focused ion beam^53^, or structured superhydrophilic and/or superhydrophobic surfaces^35^,^36^,^37^,^38^,^39^,^41^,^42^,^43^. Indeed, the patterns were completely invisible even when observed under an optical microscope, and could only be revealed via condensation which preferentially formed on the hydrophilic features (Fig. 1).

The hydrophilic features exhibited a characteristic length scale of *a* = 10 *μ*m and were shaped like circles, triangles, horizontal stripes, or vertical stripes. The pitch between features is defined as *P* = *b*/*a*, where *b* is the center-to-center separation between features. The geometry and pitch were systematically varied by fabricating arrays of pitch *P* = 2, 4, or 8 for each shape. The total width of each array is 490 *μ*m, with 250 *μ*m of space between arrays, such that the field-of-view of the microscope can simultaneously observe three different arrays (Fig. 1). For the circles, triangles, and vertical stripes, this corresponded to 25, 13, and 7 features per row for *P* = 2, 4, and 8 respectively, whereas horizontal stripes compose the entire width of their array. For simplicity, arrays will henceforth be referred to by a notation corresponding to their shape and pitch. For example, when *P* = 2, circles are C2P, triangles T2P, horizontal stripes HS2P, and vertical stripes VS2P.

### Spatial Control of Condensation

Condensation was grown on the patterned surface by cooling the substrate to either *T*_*s*_ = 5 °C (Supplementary Fig. S3) or *T*_*s*_ = −10 °C (Fig. 1) in a clean room where the ambient temperature was *T*_∞_ = 21.3 ± 0.3 °C and the relative humidity was *H* = 40 ± 3% (see Methods and Supplementary Movies 1,–6). The nucleation density of condensation is dependent upon the supersaturation of the system’s water vapor, which is typically defined as:


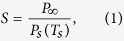


where *P*_∞_ is the partial pressure of water vapor in the ambient, while *P*_*s*_(*T*_*s*_) is the saturation vapor pressure corresponding to the temperature of the condensing surface. Here, *S* = 1.2 for *T*_*s*_ = 5 °C and *S* = 3.5 for *T*_*s*_ = −10 °C. Note that the growth of patterned condensate on the triangle arrays was nearly identical to that on the circle arrays (Supplementary Figs. S4–S6), and that the distinction between horizontal stripes and vertical stripes is only important for the later experiments involving frost growth. To avoid redundancy, discussion of patterned condensation will therefore be confined to the arrays of circles and horizontal stripes.

From classical nucleation theory^54^, the energy barrier for heterogenous nucleation of condensate is a function of surface wettability:


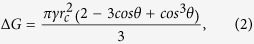


where *γ* is the liquid-vapor surface tension, *r*_*c*_ is the critical radius for stable nucleation, and *θ* is the contact angle of the nucleated droplet. Here, the receding contact angle was *θ*_*r*_ = 12° ± 2° and the advancing angle was *θ*_*a*_ = 45° ± 2° for the hydrophilic regions, while *θ*_*r*_ = 89° ± 1° and *θ*_*a*_ = 113° ± 1° for the hydrophobic regions. Assuming that a freshly nucleated droplet exhibits *θ*_*r*_ to minimize the energy barrier, Δ*G* is larger for the hydrophobic regions by a factor of ≈1,370.

For each array it was observed that every hydrophilic feature promoted the early, preferential nucleation of condensate that conformed to the shape of the feature during early droplet growth (Fig. 1). However, whether any condensation additionally nucleated on the hydrophobic background between the hydrophilic features depended upon two primary factors: the pitch of the hydrophilic patterns and the supersaturation. At *S* = 1.2, condensation exclusively nucleated on the hydrophilic patterns when *P* = 2 or *P* = 4, while a tiny minority of droplets nucleated on the hydrophobic backdrop for *P* = 8. At *S* = 3.5, spatial control was only perfect for *P* = 2, while droplet nucleation on the hydrophobic background was occurring to a very small extent for *P* = 4 and to a much larger extent for *P* = 8, such that the pattern of wetted hydrophilic features became somewhat difficult to discern. The magnitude of the dry zone was stochastic in nature; for example, at *S* = 1.2 a droplet occasionally nucleated only ≈15*μ*m from a hydrophilic feature, but at the same time, the 250 *μ*m of hydrophobic space between arrays tended to remain almost completely dry (Supplementary Fig. S3). Assuming that the lower extreme of dry zone lengths is influenced by minor chemical or physical defects (evidenced by the tendency of the nearest condensate to appear in the exact same place over multiple trials), an order of magnitude approximation of the dry zone length about a hydrophilic feature in the absence of defects is at least ~100 *μ*m for *S* = 1.2 and scales as ~10 *μ*m for *S* = 3.5.

The dry zone on the hydrophobic surface can be modeled as a competition between the system’s in-plane and out-of-plane vapor pressure gradients. These pressure gradients could facilitate a dry zone in two different ways: by inhibiting the nucleation or by inhibiting the growth of liquid condensate. Let’s consider both in turn, beginning with an inhibited-nucleation dry zone of critical length *δ*_*IN*_. From classical nucleation theory^1^,^54^, the supersaturated vapor pressure required to overcome the free energy barrier for liquid nucleation on the hydrophobic surface can be estimated as *P*_*n*_ = 2,330 Pa for *T*_*s*_ = 5 °C and *P*_*n*_ = 882 Pa for *T*_*s*_ = −10 °C. Assuming an isolated, hemi-spherical droplet filling a hydrophilic feature (*r* = 5 *μ*m), there is a hyperbolic pressure distribution, ∇^2^*P* = 0, extending radially from the droplet’s saturated interface (*P*_*s*_ = 873 Pa for *T*_*s*_ = 5 °C and *P*_*s*_ = 286 Pa for *T*_*s*_ = −10 °C^25^) toward the ambient (*P*_∞_ = 1,014 Pa). The critical distance from the perimeter of the droplet to where nucleation can first occur on the hydrophobic surface is *δ*_*IN*_ = 22.5 *μ*m for −10 °C (nucleation is not predicted to occur at all on the hydrophobic surface at 5 °C).

Even when the vapor pressure is sufficient for nucleation to occur on the hydrophobic surface (at -10° C), it is possible that the nucleated droplet will simply evaporate under certain conditions. Nucleating embryos are typically ~1 nm in size^54^. For droplets exhibiting nanometric radii of curvature, the vapor pressure is significantly modified by the Kelvin-Laplace Equation:


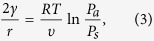


where *r* is the radius of curvature of the droplet, *R* is the universal gas constant, *T* ≈ *T*_*s*_ and *υ* are the temperature and molar volume of the water droplet, and *P*_*s*_ and *P*_*a*_ are the saturated and actual vapor pressures about the droplet. Consider an isolated *r* = 10 nm hemi-spherical droplet that has recently nucleated on the hydrophobic surface and is situated near a wetted hydrophilic feature on either side. The vapor pressure about the droplet is *P*_*a*_ = 325 Pa at *T*_*s*_ = −10 °C, such that *P*_∞_ > *P*_*a*_ > *P*_*s*_. Thus, even when nucleation is possible, the region will remain microscopically dry if the in-plane gradient driving evaporation exceeds the out-of-plane gradient facilitating condensation. The out-of-plane mass flow rate of vapor into the droplet scales as 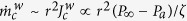, where 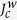 is the mass flux and *ζ* is the concentration boundary layer thickness, while the in-plane (evaporation) mass flow rate gives 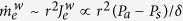, where *δ* is the edge-to-edge distance from the droplet to a wetted hydrophilic feature. The critical length scale of the inhibited-growth dry zone, *δ*_*IG*_, can be estimated by balancing the evaporation and condensation rates, 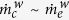
27, which gives:


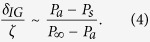


For diffusive transport, the concentration boundary layer scales as:


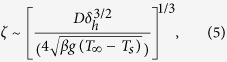


where *D* is the diffusion coefficient of water vapor, 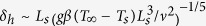 is the hydrodynamic boundary layer, *β* and *ν* are the volumetric thermal expansion coefficient and kinematic viscosity of air, and *L*_*s*_ ≈ 1 cm is the characteristic length of the condensing surface^55^. This yields *ζ* ≈ 0.8 mm and *δ*_*IG*_ ≈ 45 *μ*m for −10 °C.

Therefore *δ*_*IG*_ > *δ*_*IN*_ for the conditions used in the present experiments at –10 °C, such that nanometric condensate is able to nucleate within the majority of the dry zone, but subsequently evaporates. For *T*_*s*_ = –10 °C, the predicted length of the inhibited-growth dry zone (*δ*_*IG*_ ≈ 45 *μ*m) agrees with the experimental trend (~10 *μ*m).

### Growth of Patterned Condensation

**Figure 2:**
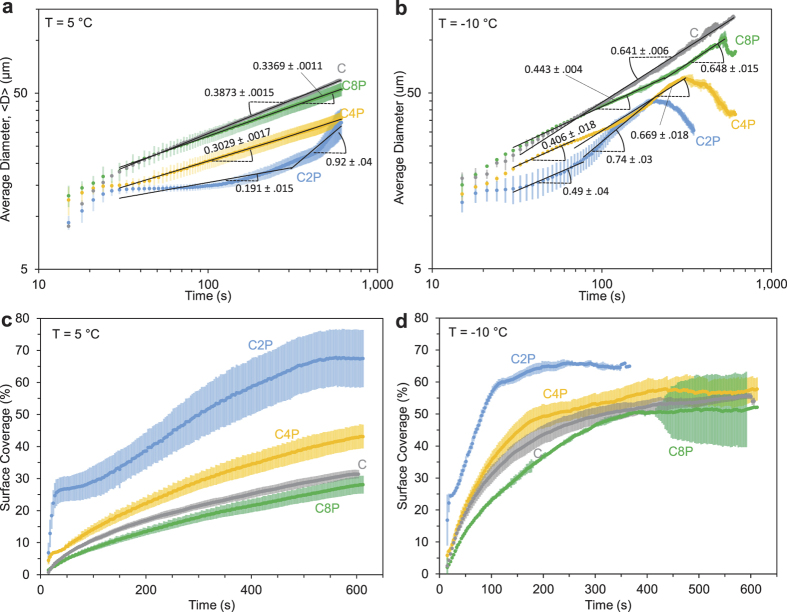
Growth of condensation on hydrophilic circle arrays of varying pitch (C2P, C4P, and C8P) or a uniformly hydrophobic control surface (C). (**a,b**) Average diameter of condensation growing at a supersaturation of *S* = 1.2 (*T*_*s*_ = 5 °C) or *S* = 3.5 (*T*_*s*_ = −10 °C), respectively. Slopes of trendlines correspond to the power law exponent *α* (Eq. (6)) within a 95% confidence interval. (**c,d**) Projected surface coverage of condensation at *S* = 1.2 or *S* = 3.5, respectively. For all plots in this section, data points correspond to an average of three trials and all error bars correspond to a standard deviation.

The average diameter (<*D*>) and surface coverage 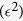 of condensate growing on the circle arrays were quantitatively extracted from the microscopy videos using ImageJ and compared to a control surface (C) that was uniformly hydrophobic (Fig. 2). On homogenous surfaces, it is now well established that dropwise condensation exhibits a power-law growth:


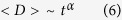


where isolated droplet growth follows *α* ≈ 1/3 and *α* ≈ 1 once regular coalescence events occur at a near-constant surface coverage (i.e. self-similar growth) of 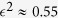
55,56. Note that <*D*> was only measured for *t* ≥ 30 s to ensure that the substrate temperature had finished cooling to its steady-state temperature.

For *S* = 1.2, *α* = 0.3873 for C (Fig. 2a), which is slightly higher than *α* ≈ 1/3 most likely due to the onset of some early (but not self-similar) coalescence events. No coalescence events occurred in the recorded time range (600 s) for C8P, and the resulting *α* = 0.3369 is in perfect agreement with the *α* ≈ 1/3 rule for isolated growth. Interestingly, the isolated growth of condensate followed *α* = 0.3029 for C4P and *α* = 0.191 for C2P, the latter of which is significantly lower than the *α* ≈ 1/3 rule for homogeneous surfaces. Compared to the control surface (C), the nucleation density was observed to be about 3 times larger for C4P and 13 times larger for C2P (Supplementary Fig. S7); we hypothesize that at low supersaturations, this dramatic increase in nucleation density caused by the hydrophilic features can increase the thickness of the water vapor concentration boundary layer, resulting in the reduced growth rate. For *S* = 1.2, only the C2P surface was able to reach a plateau surface coverage within the 600 s of recording; the growth rate of *α* = 0.92 is in good agreement with the *α* ≈ 1 rule.

For *S* = 3.5, the isolated growth of condensate on the patterned surfaces ranged from 0.4 < *α* < 0.5 (Fig. 2b), somewhat larger than the *α* ≈ 1/3 rule. It is therefore likely that hydrophilic patterns serve to enhance the growth rate of isolated condensation at high supersaturations, as the patterns serve to funnel the vapor diffusing toward the surface almost exclusively toward the hydrophilic regions. In other words, hydrophilic patterns can either enhance (by funneling vapor) or diminish (by increasing the boundary layer) the growth rate of condensation, depending upon the extent of supersaturation. However, even with the enhanced growth rates, it should be noted that the patterned surfaces exhibited smaller values of <*D*> compared to the control surface because the nucleation events were synchronized to minimize the variance in droplet volumes. At later times, where coalescence events were occurring at a steady-state surface coverage, 0.6 < *α* < 0.75. While this is lower than the theoretical trend of *α* ≈ 1, experimental measurements often hew closer to *α* ≈ 0.75; this discrepancy could be attributed to the presence of non-condensable gas, an over-simplified assumption of constant vapor flux, or the smallest droplets not being visible under the microscope objective^56^. After several minutes of coalescence-induced growth on the patterned surfaces, the value of <*D*> actually decreased due to the coalesced droplets re-exposing many of the hydrophilic features to promote fresh nucleation events. This is in sharp contrast to condensation on the uniformly hydrophobic regions (cf. hydrophobic region between C2P and C4P in Fig. 1 at 600 s), where dry zones exist about the larger droplets^34^,^57^ due to inhibited nucleation and/or growth as modeled in the previous section.

Surface coverage generally varied as 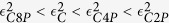 (Fig. 2c,d). While C2P, C4P, and C8P all exhibited smaller <*D*> than C, only C8P was able to achieve a smaller 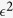 than C. This observation reveals that chemical patterns are not very effective for minimizing surface coverage relative to a uniformly hydrophobic surface, as the benefit of the hydrophilic patterns keeping the hydrophobic background dry is offset by the enhanced nucleation and growth of condensation on the hydrophilic regions. Indeed, the plateau value of surface coverage for C2P was 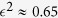 (Fig. 2d), which is larger than the typically observed value of 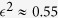 for a uniform hydrophobic surface.

**Figure 3:**
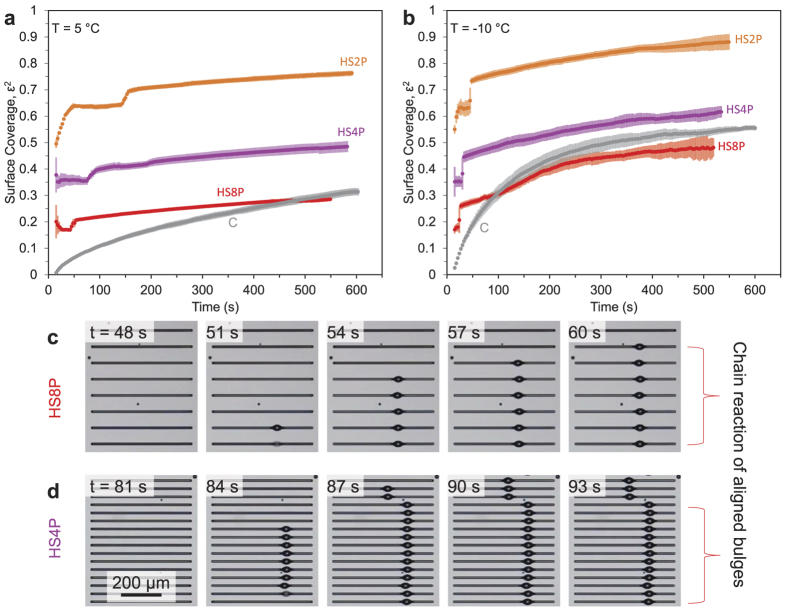
Growth of condensation on patterns of hydrophilic stripes of varying pitch (HS2P, HS4P, and HS8P) compared to a uniformly hydrophobic control surface (C). (**a,b**) Surface coverage versus time for supersaturations of *S* = 1.2 (*T*_*s*_ = 5 °C) or *S* = 3.5 (*T*_*s*_ = −10 °C), respectively. (**c,d**) When a hydrophilic stripe overflows with water and bulges out a single droplet, neighboring stripes proceed to bulge out droplets at the same axial location in a chain reaction. Images depict chain reactions occurring for HS4P or HS8P at *S* = 1.2. Since more dilute patterns of stripes are able to collect water at a faster rate than denser patterns, it follows that droplet bulging occurs first for HS8P, followed by HS4P and finally HS2P.

While the concept of <*D*> does not translate to the striped patterns, the surface coverage of HS2P, HS4P, and HS8P was analyzed (Fig. 3a,b). It was observed that 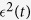 exhibited dramatically different behavior for the arrays of stripes compared to the arrays of circles or the control surface. First, condensation that nucleated within each hydrophilic stripe almost immediately spread out to cover the entire area of the stripe, resulting in very large plateau values of 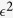 (particularly for HS2P) at unusually early time scales (*t* < 60 s). This initial plateau value of 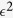 was eventually disrupted by a sudden jump to a slightly larger plateau value, which corresponds to the moment where the growing volume of water can no longer be confined entirely within the hydrophilic stripe (i.e. the contact angle of the water at the borders of the stripe reaches its advancing value). It can be observed that the water overflow from each stripe is always managed in the form of a single droplet spontaneously bulging out, in order to minimize the system’s surface energy^45^,^58^.

While the exact location along a stripe where a droplet bulges out seems be random for the first stripe where overflow occurs, a fascinating chain reaction follows where adjacent stripes proceed to bulge out droplets at the exact same location (Fig. 3c,d). In other words, the formation of a bulge in one stripe causes the preferential condensation of water on adjacent stripes in the region closest to that bulge, resulting in the overflow and formation of a bulge at the same axial location. It was observed that this chain reaction occurs more rapidly for denser patterns due to the closer proximity of the bulging droplet to the neighboring stripes; for example, in Fig. 3 a chain of seven droplets forms approximately 4 times faster for HS4P compared to HS8P. To our knowledge, this is the first time that a chain reaction of aligned droplets bulging out from adjacent stripes of water has been observed during condensation. It is an open question as to what is driving this phenomenon. The bulge configuration exhibits a smaller mean curvature compared to a non-bulged stripe of water^58^; therefore one possibility is that the bulged droplet exhibits minimal interfacial resistance and capillary resistance for condensation heat transfer^54^, which serves to maximize the flow and concentration of water vapor in the region of the bulge. Note that while the conduction resistance would be larger in the bulged droplet compared to a stripe of water, this resistance is expected to be negligible for the micrometric condensate observed here^59^. Another possibility is that inertial effects from the initial bulging of a droplet could induce a net flow in the vapor field toward the neighboring stripes. Assume that a bulging droplet exhibits a capillary-inertial velocity of 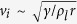 during its expansion, where *ρ*_*l*_ is the density of liquid water and *r* ~ 10 *μ*m is the radius of the bulge protruding from the stripe of water. The Reynolds number of the air then scales as 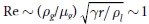 (where *ρ*_*g*_ and *μ*_*g*_ are the density and viscosity of the air/vapor surrounding the water), which might be sufficient to induce a vapor flow toward the neighboring stripes.

**Figure 4:**
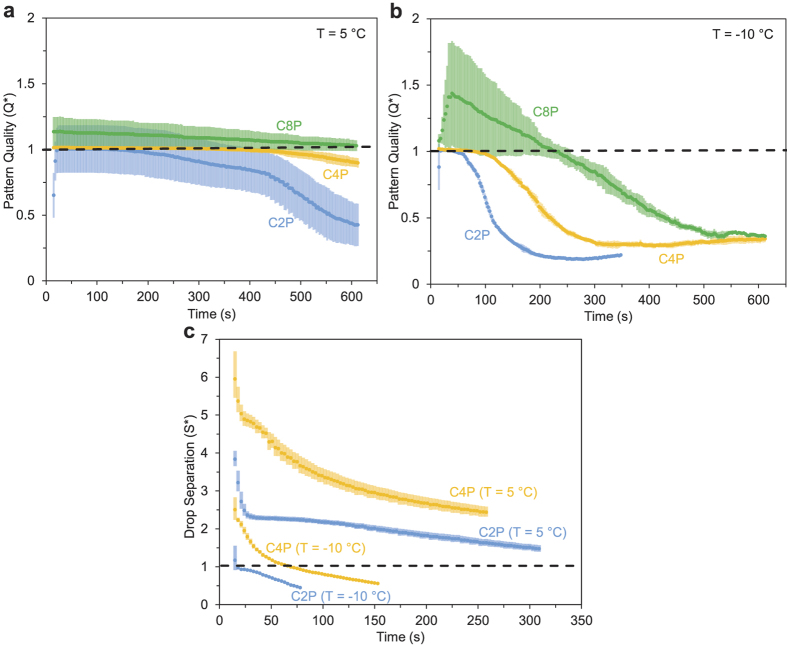
Characterizing the spatial control of patterned condensation. (**a,b**) The quality, Q^*^(*t*), represents the ratio of condensed droplets to the number of hydrophilic features (Eq. 7). (c) The drop separation coefficient, S^*^(*t*), relates the size of condensate to the inter-droplet separation (Eq. 8); for times where S^*^ > 1, inter-droplet frost growth is expected to be suppressed.

To date, very little is known regarding the ability of patterned substrates to spatially manage condensate over a period of time. In addition to measuring <*D*> and 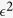, we therefore introduce two additional metrics to better quantify the spatial control of condensation on wettability patterns. A dimensionless measure of the quality of spatial control, Q^*^(*t*), is given by:


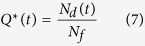


where *N*_*d*_(*t*) is the number of condensed droplets and *N*_*f*_ is the number of hydrophilic features within the same surface area. For a low supersaturation (*S* = 1.2), initially Q^*^ ≈ 1 for C2P and C4P, followed by Q^*^ < 1 upon the onset of coalescence (Fig. 4a). An advantage of the C4P array is that coalescence occurred at a later time compared to C2P, such that Q^*^ ≈ 1 for approximately 500 s rather than only 100 s. Conversely, Q^*^ > 1 at early times for C8P, due to the nucleation of droplets on the hydrophobic regions between the dilute hydrophilic features. Results were qualitatively similar for *S* = 3.5, except that C4P exhibited an extremely small degree of condensation on hydrophobic regions (Q^*^ ≈ 1.02 at early times) and the reduced quality of C8P was more exaggerated (up to Q^*^ ≈ 1.44 compared to Q^*^ ≈ 1.14 for *S* = 1.2).

Finally, when spatially controlling condensation for the express purpose of tuning inter-droplet frost growth, the most relevant metric is the separation between the droplets relative to the size of the droplets. Previously, we have demonstrated that a dimensionless droplet separation coefficient, S^*^~*L*/*D*, predicts with high accuracy whether a frozen droplet is able to grow a frost bridge to a neighboring supercooled liquid droplet of initial diameter *D* and distance *L* from the frozen droplet^21^. This scaling model is based off the observation that an ice bridge requires a two-dimensional area of roughly 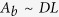 to connect to a neighboring supercooled droplet with an initial (prior to harvesting) area of 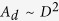. Therefore, when S^*^ > 1, the liquid droplet being harvested will completely evaporate before the ice bridge accumulates enough mass to connect, resulting in the stoppage of localized in-plane frost growth^21^. For the random growth of dropwise condensate on a homogeneous surface, measuring S^*^ requires either tedious manual measurements of *D* and *L* for every droplet^21^ or a complex image analysis program. For condensation spatially controlled by chemical micropatterns, on the other hand, the inter-droplet distance can now be easily calculated:


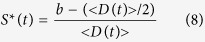


which applies to every droplet within the pattern, provided that Q^*^(*t*) ≈ 1. Note that *L* is signified by (*b* − (<*D*(*t*)/2)) because the longest possible ice bridge is when the liquid droplet has almost entirely evaporated, which adds the radius of the droplet to the total length of the connecting bridge. It can be observed in Fig. 1 that *D* and *L* are approximately equal for all droplets at a given time for the C2P and C4P patterns (prior to coalescence events), with the minor exception of droplets at the edge of the pattern which have fewer hydrophilic neighbors and are therefore slightly larger. By plotting S^*^(*t*) vs. *t*, it was observed that *P* and *S* both played significant roles regarding the length of time where S^*^(*t*) > 1 (Fig. 4c). Indeed, the duration of S^*^(*t*) > 1 was extended by over an order of magnitude for *S* = 1.2 compared to *S* = 3.5, and by several times for C4P compared to C2P at the same supersaturation. These findings have important ramifications for the subsequent growth of frost over supercooled patterns of condensation.

### Observation of Non-Local Frost Growth

**Figure 5:**
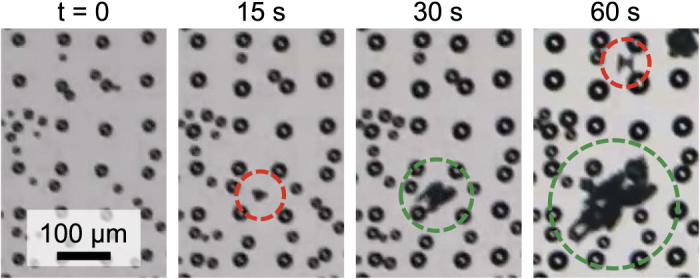
Observation of the ice shrapnel effect. Supercooled condensation growing on T8P (1^*st*^ frame) is disrupted by ice shrapnel thrown from a freezing droplet (off screen) onto a dry region of the surface (2^*nd*^ frame). The ice shrapnel proceeds to freeze neighboring droplets via inter-droplet ice bridging (3^*rd*^ frame) and subsequent freezing events eject more ice shrapnel onto the surface (4^*th*^ frame). Time zero corresponds to reaching a steady-state temperature of *T*_*s*_ = −10 °C (*S* = 3.5), red circles indicate the initial appearance of ice shrapnel, and green circles denote the subsequent inter-droplet growth. See Supplementary Movie 7.

The growth of frost was characterized at *T*_*s*_ = −10 °C for chemical patterns of pitch *P* = 2 or *P* = 4, such that the quality of spatial control for the supercooled condensate was Q^*^ ≈ 1. When the atmospheric conditions were kept identical to the previous experiments characterizing condensation (*T*_∞_ = 21 °C and *H* = 40 %), inter-droplet frost growth could not be measured repeatably due to an unexpected second mechanism of frost growth. In addition to the expected ice bridges that were grown locally from frozen droplets to neighboring liquid droplets, a surprising long-range freezing mechanism was consistently observed where a piece of ice suddenly appeared on a region of the surface far away from any pre-existing sources of ice (Fig. 5). Once these ice crystals suddenly appeared on the surface, they were able to grow in size by harvesting water from nearby liquid droplets until connecting to them, in a manner henceforth identical to locally grown ice bridges. We hypothesize that these non-local appearances of ice crystals are deposits ejected from the explosive freezing of supercooled droplets somewhere on the surface. This hypothesis of “ice shrapnel” is deduced from the following observations: (1) the region of the surface where the ice crystal suddenly appeared was often completely dry just a moment prior, ruling out the possibility of supercooled water nucleating into ice; (2) these non-local ice crystals were exclusively and abundantly observed to appear in the seconds after the freezing of a droplet(s) elsewhere on the surface, and never occurred prior to the first freezing event even over a span of several minutes; (3) the explosive freezing of supercooled water droplets has been previously observed and identified as a mechanism for increasing the amount of ice particles in clouds^60^,^61^. Note that this “ice shrapnel” effect, which was observed on a highly conductive substrate (*k*_*Si*_ ≈ 150 W/m · K) and ejects ice far away from the mother droplet, would appear to be fundamentally different from the recently reported “frost halo” effect, which was exclusive to a highly insulating surface (*k*_*PMMA*_ ≈ 0.19 W/m · K) and involves the localized evaporation and re-freezing of water vapor around the mother droplet^62^.

For the present study, it was found that the rate of frost growth was inconsistent when the effects of localized inter-droplet ice bridging were combined with the non-local ice shrapnel effect (Supplementary Fig. S8). Interestingly, it was observed that when the same experiments were repeated in a different laboratory under less humid conditions (*T*_∞_ = 24.0 ± 0.9 °C, *H* = 26 ± 2%, *S* = 2.7), the ice shrapnel effect never occurred. It is likely (but not certain) that the decrease in humidity was directly responsible for the disappearance of the ice shrapnel, as this is in agreement with a previous report that shattering did not occur for droplets freezing at sufficiently cold (and therefore less humid) air temperatures^60^. Therefore, all experiments in the proceeding sections were performed at these new environmental conditions, such that localized ice-bridging was isolated as the sole mechanism of inter-droplet frost growth.

### Tuning Inter-Droplet Frost Growth

**Figure 6:**
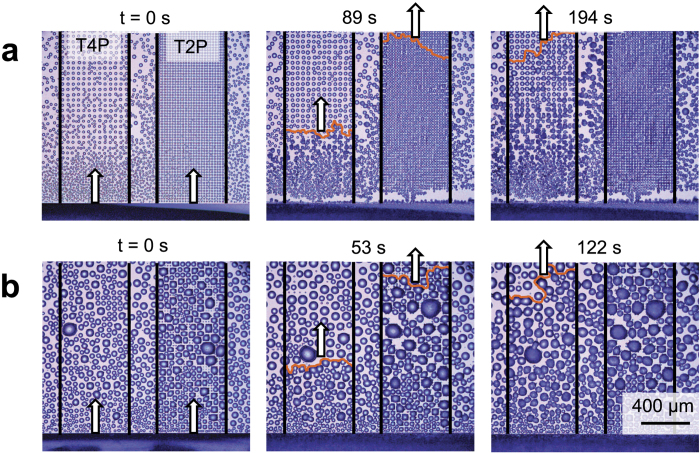
Inter-droplet frost growth across patterns of supercooled condensation (T4P on left and T2P on right). Freezing was initiated **(a)** 1 min or **(b)** 5 min after reaching a steady-state temperature of *T*_*s*_ = −10 °C (*S* = 2.7) by touching a piece of ice to a rectangular film of water (bottom of the images) bordering the droplet arrays. In each figure, the 1^*st*^ frame shows the onset of freezing (called time zero), while the 2^*nd*^ and 3^*rd*^ frames represent the times where the inter-droplet frost grew to the top of the field-of-view in the T2P and T4P arrays, respectively. Measured velocities of frost growth were **(a)** *v*_*T*4*P*_ = 4.38 *μ*m/s and *v*_*T*2*P*_ = 10.01 *μ*m/s; **(b)** *v*_*T*4*P*_ = 6.05 *μ*m/s and *v*_*T*2*P*_ = 16.73 *μ*m/s. See Supplementary Movies 9 and 10.

The separation between droplets (S^*^) exhibits a strong dependence upon both the pitch of the chemical pattern and the time allowed for condensation to grow (Fig. 4c); therefore, it is now clear that the onset of freezing must be temporally controlled to properly tune the rate of inter-droplet frost growth. To control both the time and location at which freezing first occurred on the surface, a large hydrophilic rectangle was patterned along the edge of each silicon chip, such that a thin film of water could be deposited onto the surface that bordered one end of the chemical arrays. Regardless of the pitch or shape of the chemical arrays, the edge-to-edge separation between the water film and the first row of patterned features was 10 *μ*m. Once the edge of the wafer was primed with this thin film of water, the surface was cooled beneath the dew point to a subfreezing temperature in order to grow the patterned condensation as usual. At the desired time, the supercooled film of water was frozen by gently touching a small piece of ice to the edge of the wafer, and frost proceeded to propagate across the arrays of chemical patterns via inter-droplet ice bridges (Fig. 6, Supplementary Figs S9–S11). In this manner, the patterned arrays of supercooled condensation now resemble race tracks (see Supplementary Movies 9,10,11,12,13–14), where the propagation speed of frost growth can be characterized as a function of the pattern geometry and the time elapsed before the onset of freezing.

**Figure 7:**
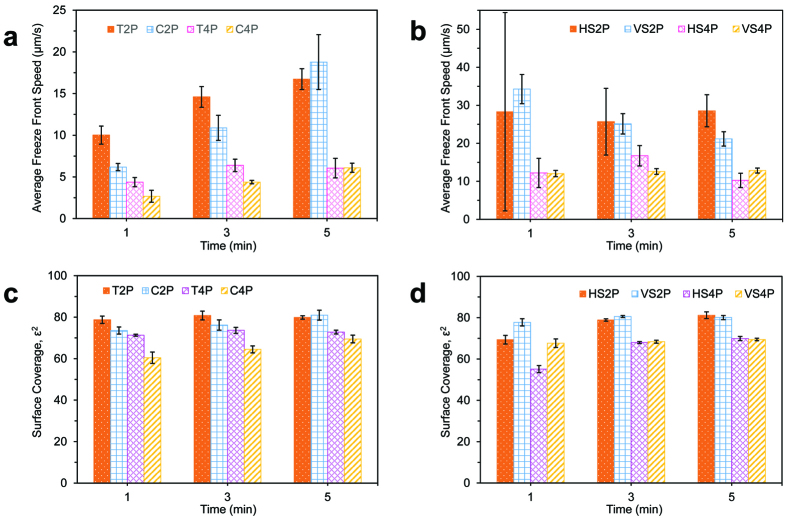
Propagation velocity (*v*) and surface coverage (*ε*^2^) of frost growing over various patterns of supercooled condensation at *T*_*s*_ = −10 °C and S = 2.7. (**a,b**) The average velocity of frost growth could be tuned by the geometry and spacing of the patterns and also depended upon the time of the initial freezing event. (**c,d**) After the pattern of condensation visible in the field-of-view had completely frozen over, the surface coverage of frost was measured; *ε*^2^ was approximately 0.2 times lower for the *P* = 4 patterns compared to the *P* = 2 patterns.

The average speed of inter-droplet frost growth across supercooled condensation patterns was measured for arrays of circles (C2P/C4P), triangles (T2P/T4P), and stripes running parallel (HS2P/HS4P) or perpendicular (VS2P/VS4P) to the frozen water film (Fig. 7). In each trial, the deposited film of water adjacent to the arrays was frozen either 1, 3, or 5 min after the surface reached a steady-state temperature of *T*_*s*_ = −10 °C. The velocity of the inter-droplet freezing front is expressed as 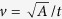, where *A* is the total area enclosing a particular array and *t* is the time required for frost to completely grow over *A*. Depending upon the shape and pitch of the array and the time of initial freezing, the value of *v* could vary by at least an order of magnitude. As expected, frost was able to grow faster when *P* = 2 compared to *P* = 4, due to the closer proximity of the nucleation sites greatly decreasing the value of S^*^ (cf. Fig. 4c). The values of *v* increased with later times of freezing onset for arrays of circles or triangles, and the extent of the difference in *v* between *P* = 2 compared to *P* = 4 also became amplified at later freezing times. In contrast, the freezing time was not nearly as important for the striped patterns, as the surface coverage of the stripes reaches a plateau value at extremely early time scales and is not as sensitive to time (cf. Fig. 3). Furthermore, the average speed of the frost growth was much more inconsistent for the HS patterns, as it could vary widely depending upon how many of the bulged droplets were axially aligned to minimize the spacing between each body of water. Note that the geometry of the chemical patterns not only controls the velocity of frost growth, but also the macroscopic shape of the ice. This was characterized in terms of the surface coverage of the frost, which is defined as the fraction of the projected area that contains ice. Recall that the surface coverage of supercooled condensation at any given time was smaller for *P* = 4 arrays compared to *P* = 2; since in-plane frost growth is essentially an inter-droplet phenomenon, it therefore follows that the surface coverage of the frost is also lower for *P* = 4. In short, both the speed and surface coverage of in-plane frost growth is dependent upon S^*^, which in turn can be tuned by chemical patterns and controlling the time of initial freezing.

### Halting Inter-Droplet Ice Bridging

**Figure 8:**
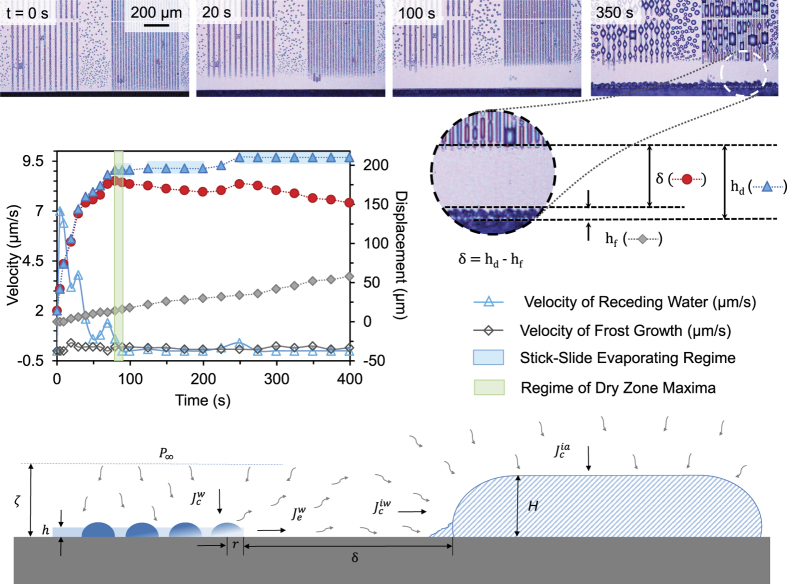
Demonstration of halted inter-droplet frost growth. By freezing the film of water (visible at the bottom of the pictures) immediately after cooling down to *T*_*s*_ = −5 °C, the size of the water droplets was sufficiently small to prevent the success of ice bridging. Even when the substrate was subsequently cooled to *T*_*s*_ = −12.5 °C, the surface area adjacent to the frozen film remained dry for over 5 min The dry zone would have lasted even longer, but frost eventually invaded the surface from the side, due to frost propagation from an uncontrolled region of the surface. See Supplementary Movie 15. The schematic illustrates the dry zone between ice and the condensate; the border of the dry zone corresponds to the point where the condensation and evaporation rates of the supercooled droplets are perfectly balanced.

In the previous experiments, where freezing was initiated either 1, 3, or 5 min after cooling to *T*_*s*_ = −10 °C, the frost was always able to grow across the surface. This is because as the surface is cooled to *T*_*s*_ = −10 °C, S^*^ > 1 is only true for roughly 20 s of condensation growth for *P* = 2 patterns and 60 s for *P* = 4, including the 30 s required for the cooling transient (cf. Fig. 4c). Therefore, to completely stop inter-droplet frost growth via ice bridging, the onset of freezing needs to occur as early as possible, such that *S*^∗^ > 1 and water droplets will completely evaporate before the ice bridges can connect. To demonstrate that inter-droplet ice bridging can be completely halted, a surface exhibiting VS2P and VS4P patterns was frozen immediately after reaching *T*_*s*_ = −5 °C and then cooled all the way down to *T*_*s*_ = −12.5 °C. The water contained in the stripes proceeded to evaporate and recede away from the ice, creating a dry zone that reached a maximal value of approximately *L* = 180 *μ*m that was stable for over 5 min (Fig. 8). This would appear to be the first report of halting inter-droplet ice bridging between ice and its neighboring supercooled condensate. While one recent report did observe the inability of ice to connect to supercooled condensate on a hydrophobic surface^24^, the ice was isolated at an edge of the substrate at least 80 *μ*m away from the nearest condensate and the dry zone was only monitored for 2 min. Here, the initial separation between the ice and condensate was a mere 10 *μ*m, and the freezing of the ice served to increase this gap by a factor of 18 for at least 5 min.

While successful in preventing the ice from bridging to neighboring condensate, a single dry zone does have two long-term limitations. Firstly, far from the dry zone, the supercooled condensation is still free to grow, and will eventually freeze and grow ice bridges across the surface. This was evident in Fig. 8, where the dry zone itself was stable but frost was able to invade across the droplet arrays from elsewhere on the surface. Secondly, while the condensation growing beyond the edge of the dry zone does exhibit a net influx of vapor, these droplets still exhibit a partial outflux of vapor that migrates across the dry zone and is harvested by the ice. It is for this reason that the film of ice continues to grow in-plane over time even after the dry zone has formed, as can be observed in the graph in Fig. 8. It should be emphasized that the in-plane growth of the isolated ice patch is of order *v* ~ 0.1 *μ*m/s (Fig. 8), two orders of magnitude slower than *v* ~ 10 *μ*m/s observed for traditional frost growth via inter-droplet ice bridging (Fig. 7a,b). Furthermore, the advancement of the ice serves to translate the dry zone upward, resulting in the continued evaporation and receding of the nearest water region. Sometimes, the water is not able to recede quite as quickly as the ice advances, as evidenced from the very slowly decaying dry zone length (*L*) plotted in Fig. 8. The slower receding of the water is attributed to two factors: contact angle hysteresis causing a stick-slide behavior, and the evaporating water being replenished by more water further up the patterns in the case of the stripes. Finally, it is interesting to note that the shape of the advancing ice front depends upon the geometry of the water being harvested (see Supplementary Movies 15,16–17): a fingering shape results for ice growing toward discontinuous patterns (Supplementary Fig. S12), while the ice front is more uniform when growing toward horizontal stripes of water (Supplementary Fig. S13).

The length of the dry zone about the ice, *δ*_*IG*_, can be theoretically estimated using the same flux balance approach used for calculating the condensation dry zones (see ‘Spatial Control of Condensation’ section). The pressure gradients are driven by *P*_∞_ > *P*_*s*_ > *P*_*s*,*i*_, where *P*_*s*_ and *P*_*s*,*i*_ are the saturation pressures of water and ice, respectively. The perimeter of the dry zone corresponds to the point where the net mass flux condensing into a liquid droplet from the ambient air is equal to the net mass flux evaporating from the droplet towards the ice. The condensed droplets bordering the dry zone can be approximated as an equivalent film of water of height *h*^27^, where *h* ~ *r*, *r* being the mean droplet radius (see schematic in Fig. 8). This film has at its top a pressure *P*_*s*_ corresponding to the saturation pressure of water at *T*_*s*_. Let us take a thin strip of the film of length *L* along the perimeter, height *h* and width *r*. Then the mass flow rate of vapor condensing onto the droplets due to the out-of-plane pressure gradient scales as 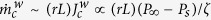. The in-plane mass flow rate of vapor evaporating out towards the ice scales as 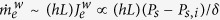. Equating the condensation and evaporation rates at the border of the dry zone:


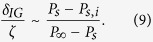


For the experimental conditions *T*_*s*_ = −12.5 °C, *T*_∞_ = 24 °C, and *H* = 26%, the corresponding vapor pressures are *P*_∞_ = 776 Pa, *P*_*s*_ = 235 Pa and *P*_*s*,*i*_ = 208 Pa. Using Eq. 9, the experimentally observed dry zone length of *δ*_*IG*_ ≈ 180 *μ*m corresponds to a concentration boundary layer thickness of *ζ* ≈ 3.7 mm. This agrees with a theoretical estimation of *ζ* using Eq. 5, where *ζ* ~ 1 mm is obtained.

## Discussion

Several recent reports on delaying frost growth with superhydrophobic surfaces have focused on maximizing the elapsed time before any freezing events occur within the population of supercooled condensation^3^,^4^,^5^. Our observations from the present study suggest rather counterintuitively that the exact opposite approach is preferable: by intentionally triggering an extremely early freezing event on the surface, the spacing between droplets is maximized to slow or even halt the inter-droplet growth of frost. Indeed, far from being undesirable, early freezing events can create dry zones, where all of the adjacent water droplets are harvested by the ice due to the localized gradient in vapor pressures (Fig. 8).

The present study characterized condensation and frost formation with the ambient environment at room temperature and fixed humidity, in contrast to natural systems where the air and surface temperatures are similar and the humidity can fluctuate widely. A recent study utilized an environmental chamber to chill the ambient air and widely vary the humidity, and confirmed that inter-droplet ice-bridging remains the primary driver of in-plane frost growth over this wider parameter space^29^. Cooler and/or drier ambient conditions would correspond to a decreased supersaturation (*S*), which would likely increase the critical pitch of the hydrophilic patterns to maintain spatial control and magnify the effects of the pressure gradient between water and ice responsible for the dry zones. Therefore, the highly supersaturated conditions used here are actually somewhat extreme; it should be easier to pattern condensation and/or prevent inter-droplet ice bridging in a more natural environment. It should also be emphasized that silane monolayers, while ideal for fundamental studies, are prone to degradation under long-term condensation or frosting conditions. More durable hydrophobic coatings, such as grafted polymers^63^, graphene^64^, or intrinsically hydrophobic materials would therefore seem more appropriate for practical applications. The surfaces used for the present study were thermally conductive and extremely smooth; it would be interesting for future studies to characterize how the spatial control of condensation and frost could be modified by practical materials that exhibit varying degrees of roughness and thermophysical properties.

In the present work, we triggered an early freezing event to locally dry out condensate and create a single dry zone on the chilled surface. While stable for several minutes at the very least, the long-term durability of the dry zone is doubtful due to the continued growth of water and ice far away from the dry zone. We suggest that a possible solution to this problem is triggering multiple freezing events simultaneously at an early time scale, for example creating an array of thin stripes of ice, such that multiple dry zones are created that overlap to permanently dry all of the liquid water away from the regions between the ice arrays. In other words, rather than patterning the supercooled condensation and triggering an isolated freezing event, it would now appear to be advantageous to pattern the freezing water itself!

In conclusion, the spatial control of liquid condensation on chemical patterns, as well as the formation of a dry zone between ice and supercooled water, are both driven by a fascinating competition between in-plane and out-of-plane gradients in vapor pressure. Smooth chemical micropatterns can spatially control both the nucleation and growth of condensation even at very large supersaturations, provided that the distance between hydrophilic features is not too large. Compared to a uniform hydrophobic surface, condensation on a chemically patterned surface exhibited smaller average diameters and more homogeneous droplet volumes and inter-droplet separations. Therefore chemically patterned surfaces serve to maximize the spacing between condensate, such that the in-plane growth of frost via inter-droplet ice bridging can be delayed or even completely halted. Since the separation between droplets decreases with increasing duration of condensation, it was demonstrated that intentionally triggering an early freezing event on the surface is also crucial for halting ice bridging. Indeed, extremely early freezing events were shown to halt inter-droplet frost growth even on the non-patterned regions of the surface. Therefore, the inter-droplet growth of frost across a surface can be minimized or even stopped by spatially and/or temporally maximizing the separation between supercooled condensate upon the onset of freezing.

## Methods

### Sample fabrication

Silicon wafers (4 in., Silicon Quest) were first cleaned with Piranha solution (3:1 H_2_SO_4_:H_2_O_2_ at 25 °C for 10 min), rinsed extensively in deionized water, and dried with nitrogen. Clean wafers were functionalized with trichloro(1H, 1H, 2H, 2H−perfluorooctyl)silane (Sigma-Aldrich) through vapor deposition in a covered glass petri dish heated to 80 °C for 1 hr. Parylene was then deposited onto the silanized wafers using 800 mg of 2,2-paracyclophane precursor (Sigma) loaded into a parylene coater (SCS Labcoter 2, Specialty Coating Systems) run under standard operating conditions. Next, MP−P20 adhesion promoter (MicroChem) and S1818 photoresist (Shipley) were respectively spin-coated onto the wafer, both at 3,000 rpm for 45 s. After a 60 s bake at 115 °C, the wafer was exposed to ultraviolet light at 10 mW/cm^2^ at 365 nm for 5 s (Quintel Mask Alignment System) and developed in CD −26 for 2 min, rinsed with deionized water, and dried with nitrogen gas.

The wafer was then etched with oxygen plasma in an inductively coupled plasma ion etching system (Oxford Plasmalab 100, Oxford Instruments) for 30 min. The etch rate under these conditions was approximately 62 nm/min, such that 30 min was sufficient to etch through the exposed parylene (1.25 *μ*m thick) and the remaining photoresist (1.75 *μ*m thick) while preserving the parylene that was underneath the photoresist. Immediately before use, the wafer was etched in oxygen plasma for 1–3 min to ensure that the exposed silicon oxide regions were hydrophilic, followed by a dry peel-off of the parylene film using tape and tweezers^52^ to expose the hydrophobic silane monolayer.

### Condensation experimental procedure

A freshly prepared silicon sample was bonded to a Peltier stage (Deben MK3 Coolstage) with thermal paste and placed under a top-down optical microscope (Nikon Eclipse LV150). The ambient temperature was *T*_∞_ = 21.3 ± 0.3 °C and the relative humidity was *H* = 40 ± 3%, corresponding to a dew point of *T*_*DP*_ = 7.2 ± 1.3 °C. The substrate was initially kept dry at 10 °C and was then cooled beneath the dew point to 5 °C to allow condensation to form in order to make the patterns visible. The desired pattern type (circles, triangles, or stripes) was centered and focused under a 5× objective lens, such that the 2P, 4P and 8P arrays were all visible within the microscope’s field-of-view.

Once the field-of-view was set, the sample was immediately heated to 40 °C to dry the surface. The wafer was then cooled back down to 10 °C, followed by further cooling to either 5 °C or −10 °C to observe the resulting condensation with a digital camera (Nikon DS-5M) attached to the microscope. The transient of the Peltier stage was 5 ± 1 s to cool from 10 °C to 5 °C and 30 ± 1 s to cool from 10 °C to −10 °C. The forming condensation was imaged for 10 min, and the surface was then heated back to 40 °C to dry out the surface for the next trial. Three trials were obtained for both condensation temperatures and a new chip was used for each pattern type to minimize surface contamination.

### Image Analysis of Condensation

A stack of images from each condensation video were analyzed in ImageJ, with a time interval of 3.0 s between each frame. For arrays of circles or triangles, there were 140, 507, and 1,925 hydrophilic features within the field of view for *P* = 2, 4, and 8 respectively. For arrays of stripes, there were 20, 39, and 77 stripes in the field of view for *P* = 2, 4, and 8 respectively. Each stack was cropped to analyze each array (2P, 4P, or 8P) in isolation. Each cropped stack was digitally sharpened and then converted to a binary mask. For the 4P and 8P arrays, the ‘Fill Holes’ process was performed to fill in holes in the droplets caused by reflected light, followed by the ‘Watershed’ process to ensure that droplets that were close to coalescing were not counted as a single droplet. The ‘Analyze Particles’ procedure was then performed to measure droplet diameters and surface coverage. For the 2P array, the droplets were too small for the ‘Fill Holes’ process to work accurately, so the following alternate approach was taken for the image processing. After sharpening the image and converting to binary, the ‘Close’ process was used to fill in the holes of the droplets, followed by ‘Erode’, ‘Watershed’, ‘Dilate’, and ‘Watershed’, respectively to separate nearby droplets from each other.

### Condensation frosting experimental procedure

Under the same atmospheric conditions as with the condensation experiments (*T*_∞_ = 21.3 ± 0.3 °C and *H* = 40 ± 1%), the desired pattern was centered and focused under the microscope by condensing water at 5 °C, followed by drying at 40 °C and holding steady at 10 °C. The field-of-view under the microscope included a large rectangular hydrophilic region that bordered the hydrophilic arrays on one edge of the wafer. A 2.5 *μ*L droplet of deionized water was deposited onto this rectangular hydrophilic pad, followed by cooling down to −10 °C to induce the growth of supercooled condensation on the hydrophilic array. The array in the field-of-view included 2P, 4P, and 8P features that were either circles, triangles, stripes running parallel to the deposited water droplet, or stripes running perpendicular to the deposited water droplet. To trigger inter-droplet ice bridging across the patterned condensation, a small piece of ice was gently touched against the side of the wafer containing the deposited water film to freeze it. To vary the rate of frost growth, the water pad was frozen at different times (0 s, 30 s, 60 s, 90 s, 120 s, or 270 s), where time zero corresponds to the instant where the surface finishes cooling to the set point temperature of −10 °C. The resulting frost growth was characterized by dividing the square root of the total area of each array by the time required for it to frost over. It should be noted that in addition to inter-droplet ice bridging, the frost growth for these experiments was also affected by ice shrapnel, where freezing droplets sprayed small particles of ice over the surface that induced non-local ice bridging events in addition to those caused by the initial freezing event.

Condensation frosting was characterized a second time in a less humid environment that suppressed the ice shrapnel, in order to isolate the kinetics of localized inter-droplet ice bridging from a single freezing event. This second set of experiments was nearly identical to the methods described above, except now *T*_∞_ = 24.0 ± 0.9 °C and *H* = 26 ± 2% corresponding to a dew point of *T*_*DP*_ = 3.3 ± 1.9 °C. The surface was initially held at 3 degrees above the dew point, and cooled down to a steady-state temperature of *T*_*s*_ = −10 °C which took approximately 30 s. The hydrophilic pad of deposited water was frozen at three different times (1 min, 3 min, or 5 min) after reaching the steady-state temperature for all 2P and 4P shapes, with three trials performed for each parameter. The resulting frost growth was imaged to measure the averaged velocity of frost growth, in an identical manner to the first set of experiments. Finally, to capture a stable dry zone (Fig. 8), the surface was initially held at the lowest possible temperature of *T*_*s*_ = 1 °C where no condensation formed; while this was below the theoretical dew point by a few tenths of a degree, this can be attributed to the small thermal resistance between the Peltier stage and the substrate or because vapor can become supersaturated at a surface without condensing due to the energy barrier for nucleation. The surface was then cooled down to *T*_*s*_ = −5 °C and the water pad was immediately frozen, to minimize the value of S^*^. Once the pad was frozen and the ice began to harvest water from the condensation, the surface was then cooled to a final temperature of *T*_*s*_ = −12.5 °C to demonstrate the long-term stability of the dry zone even under highly supersaturated conditions.

## Additional Information

**How to cite this article**: Boreyko, J. B. *et al.* Controlling condensation and frost growth with chemical micropatterns. *Sci. Rep.* **6**, 19131; doi: 10.1038/srep19131 (2016).

## Supplementary Material

Supplementary Information

Supplementary Movie 1

Supplementary Movie 2

Supplementary Movie 3

Supplementary Movie 4

Supplementary Movie 5

Supplementary Movie 6

Supplementary Movie 7

Supplementary Movie 8

Supplementary Movie 9

Supplementary Movie 10

Supplementary Movie 11

Supplementary Movie 12

Supplementary Movie 13

Supplementary Movie 14

Supplementary Movie 15

Supplementary Movie 16

Supplementary Movie 17
