# Supplementary material for: Controlling condensation and frost growth with chemical micropatterns
Source: Sci Rep. 2016 Jan 22;6:19131. doi: 10.1038/srep19131 (PMC4726256; doi:10.1038/srep19131)
Supplement: Supplementary Information [file srep19131-s1.pdf]

Supplemental Information: Controlling condensation and frost growth with chemical micropatterns

Jonathan B. Boreyko[1,2,3], Ryan R. Hansen[2,3,4], Kevin R. Murphy[1], Saurabh Nath[1], Scott T. Retterer[2,3,5], and C. Patrick Collier[2,3]

[1]Department of Biomedical Engineering and Mechanics, Virginia Tech, Blacksburg, Virginia 24061, USA
[2]Center for Nanophase Materials Sciences, Oak Ridge National Laboratory, Oak Ridge, Tennessee 37831, USA
[3]Bredesen Center for Interdisciplinary Research and Graduate Education, The University of Tennessee, Knoxville, Tennessee 37996, USA
[4]Chemical Engineering Department, Kansas State University, Manhattan, Kansas 66506, USA
[5]Biosciences Division, Oak Ridge National Laboratory, Oak Ridge, Tennessee 37831, USA

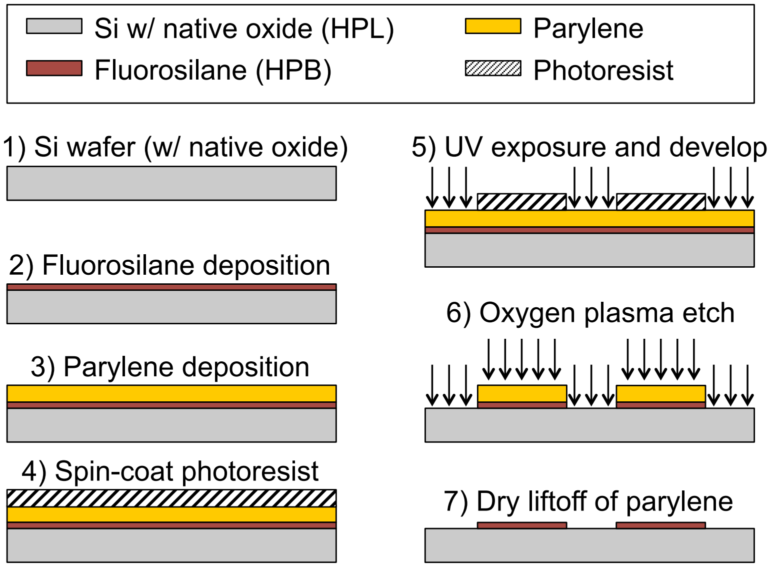

Supplementary Figure S1: Schematic of the procedure for the microfabrication of the chemical patterns. See Methods in the main text for more detailed information.

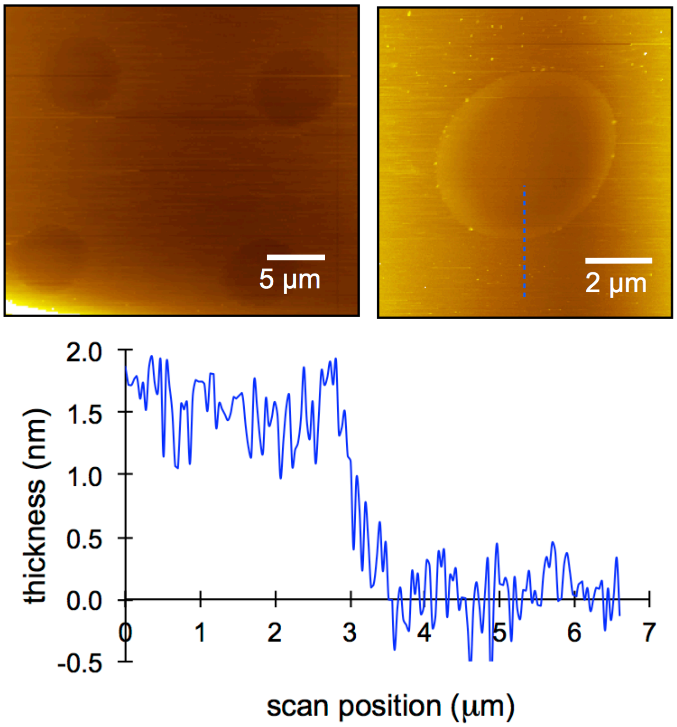

Supplementary Figure S 2: Atomic force microscopy of the chemical micropatterns. The blue dotted line in the upper-right micrograph represents the line scan depicted below graphically. The average thickness of 1.5 nm corresponds to a self-assembled silane monolayer comprising the hydrophobic surface. Note that while the hydrophilic circles shown here are 5 μm in diameter compared to the 10 μm patterns used in the condensation/frosting experiments, the fabrication steps to create the patterns were identical.

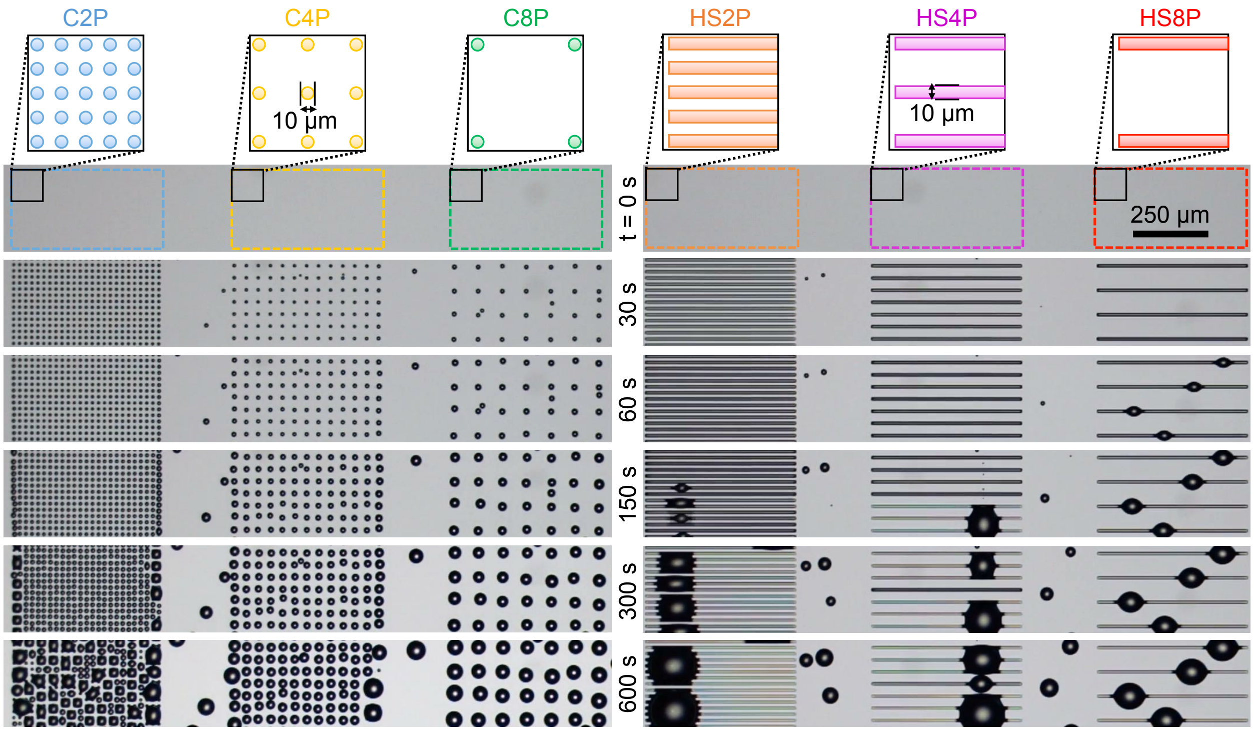

Supplementary Figure S3: Spatial control of condensation on smooth chemical micropatterns composed of arrays of circles or stripes. The colored shapes in the schematics represent the hydrophilic features while the white background is hydrophobic. Time zero corresponds to the onset of cooling from 10°C down to a steady-state temperature of $T_s = 5\,°\mathrm{C}$ from 5 s onward. See Supplementary Movies 1 and 3.

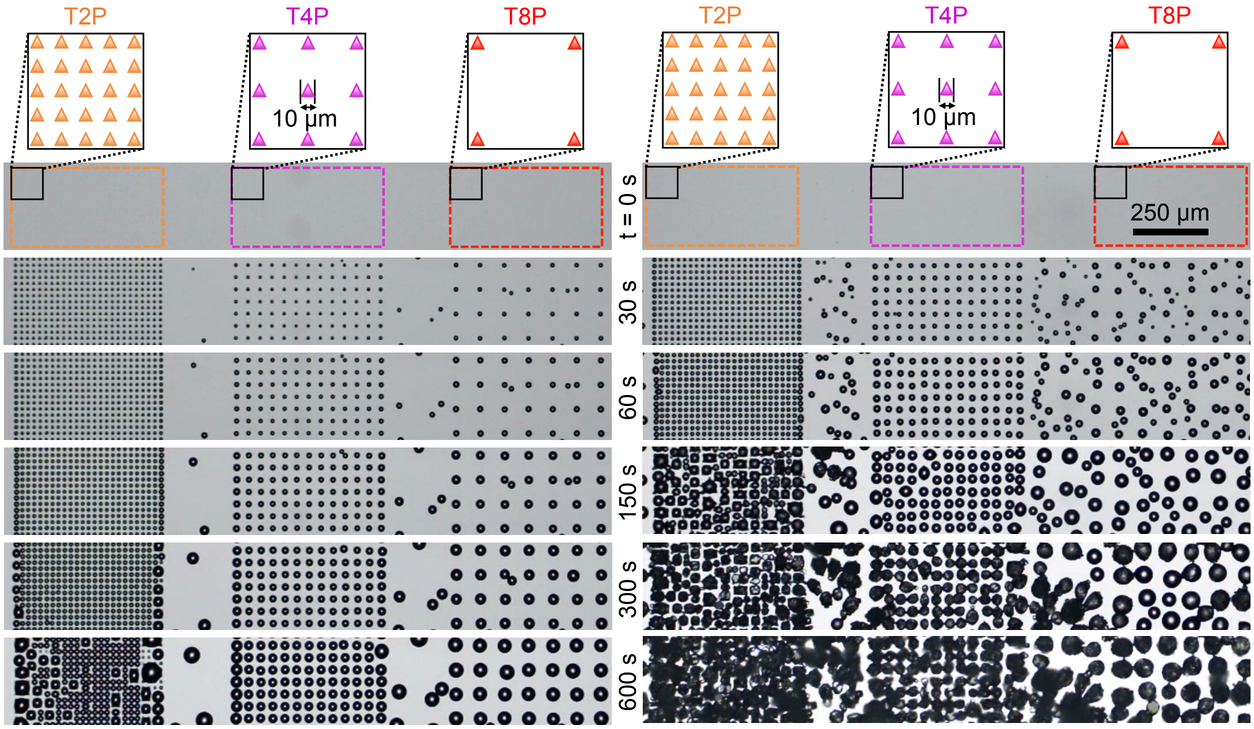

Supplementary Figure S4: Spatial control of condensation on smooth chemical micropatterns composed of arrays of hydrophilic triangles. Time zero corresponds to the onset of cooling from 10°C down to a steady-state temperature of $T_s = 5\,°C$ (images on left) or $T_s = -10\,°C$ (right). See Supplementary Movies 5 and 6.

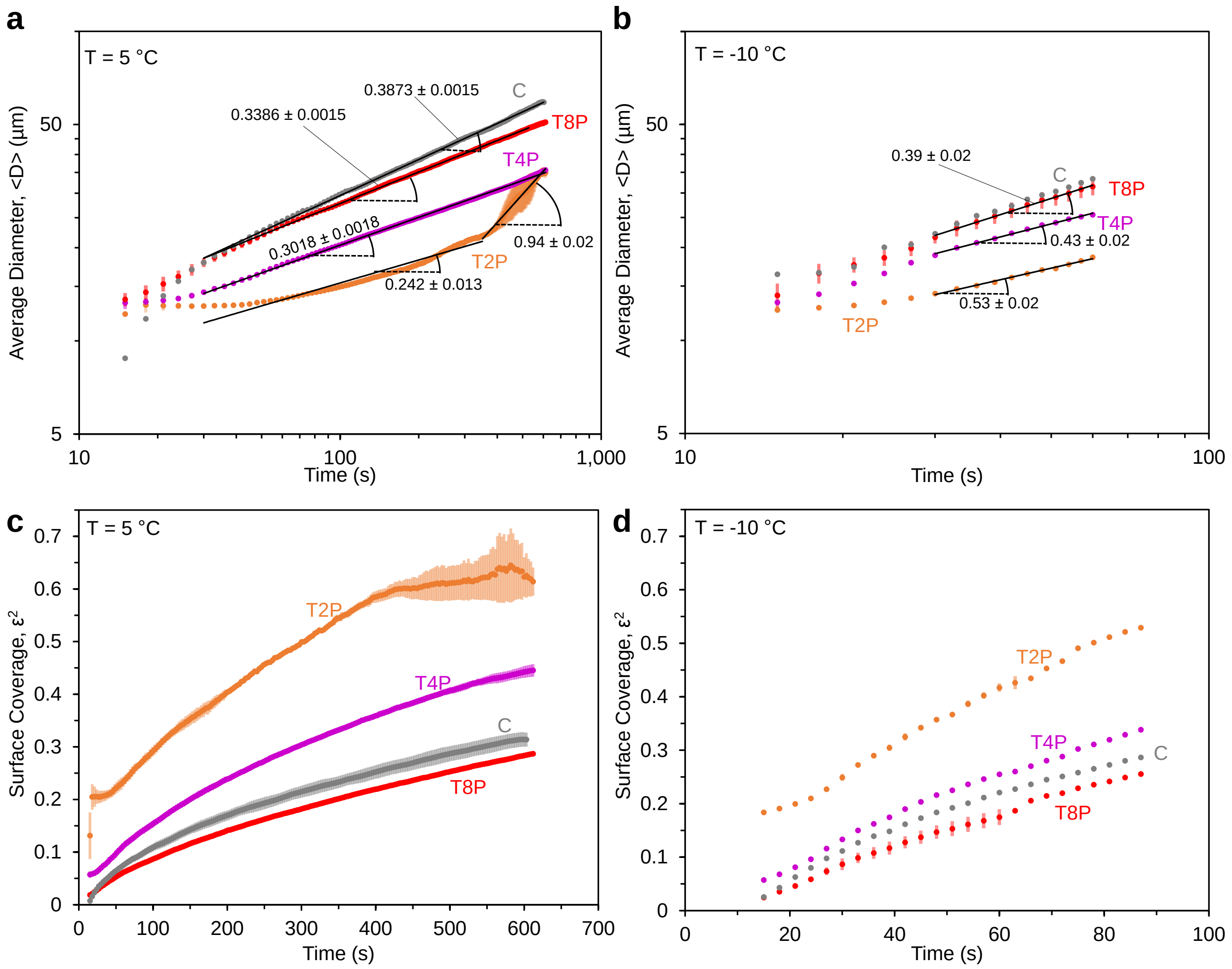

Supplementary Figure S5: Average diameter and surface coverage of condensation growing on arrays of hydrophilic triangles. (**a**) In a manner equivalent to condensation on the circle arrays (cf. Fig. 2), the growth rate of isolated condensation on T4P or T2P was smaller than the typical $\alpha \approx 1/3$ rule at a low supersaturation ($S = 1.2$). As previously discussed, this is likely due to the combination of a highly elevated nucleation density and a small supersaturation serving to increase the boundary layer of the vapor pressure gradient. (**b**) Also in a manner similar to the circle arrays, the growth rate of isolated condensate on T4P and T2P was higher than the $\alpha \approx 1/3$ rule at higher supersaturations ($S = 3.5$). Unlike with the circle arrays, heterogeneous nucleation and frost growth occurred only several minutes into the experiment, so the growth rates at a plateau surface coverage could not be measured. (**c,d**) As with the circle arrays, the surface coverage of condensate was larger for T2P and T4P compared to the uniformly hydrophobic control surface (C).

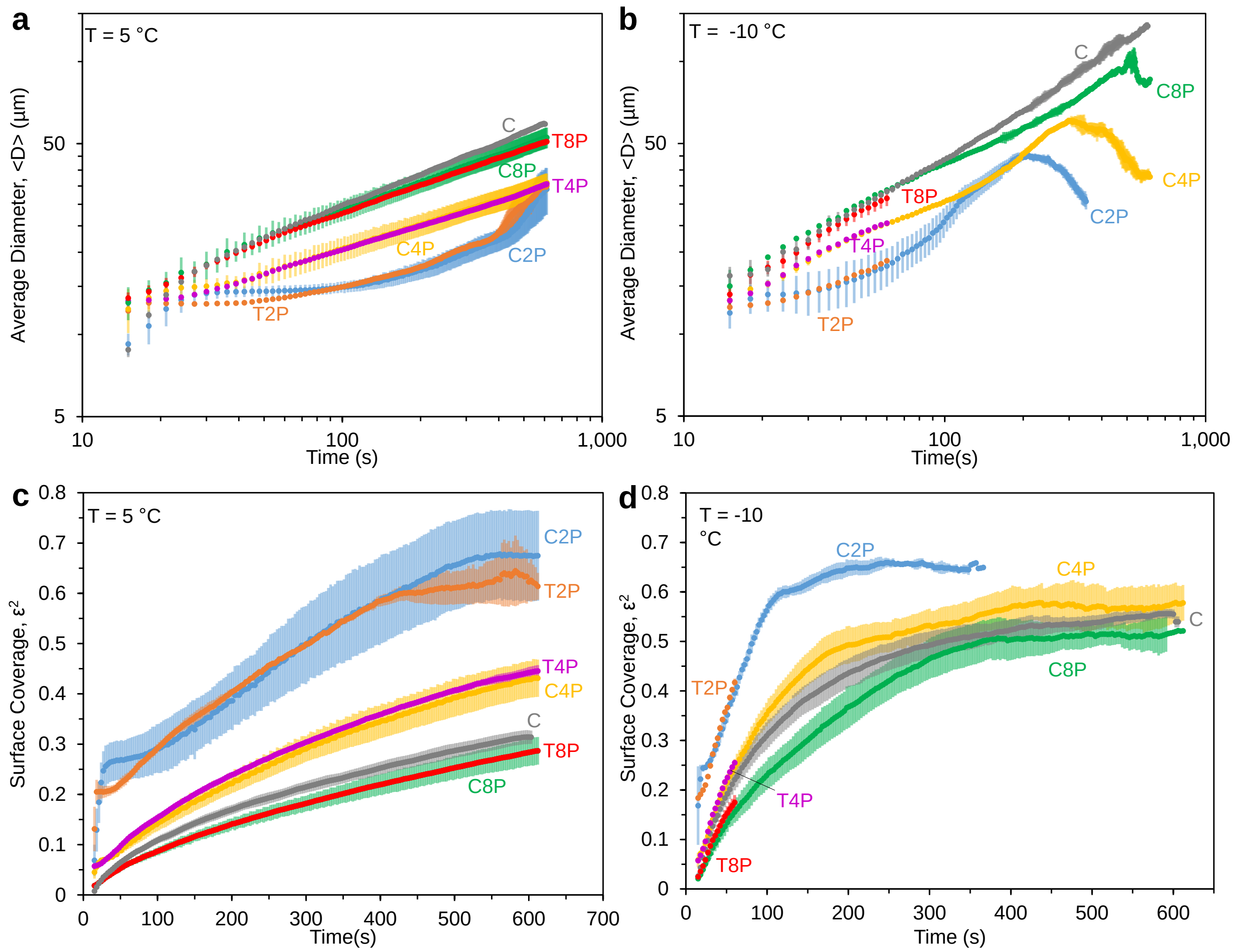

Supplementary Figure S6: A direct comparison of condensation on the circle arrays and triangle arrays (overlay of Fig. 2 and Supplementary Fig. S5). Because the water nucleating on the hydrophilic patterns tends to grow beyond the hydrophilic/hydrophobic borders within a few seconds, the condensation growth on the circles versus triangles is essentially equivalent.

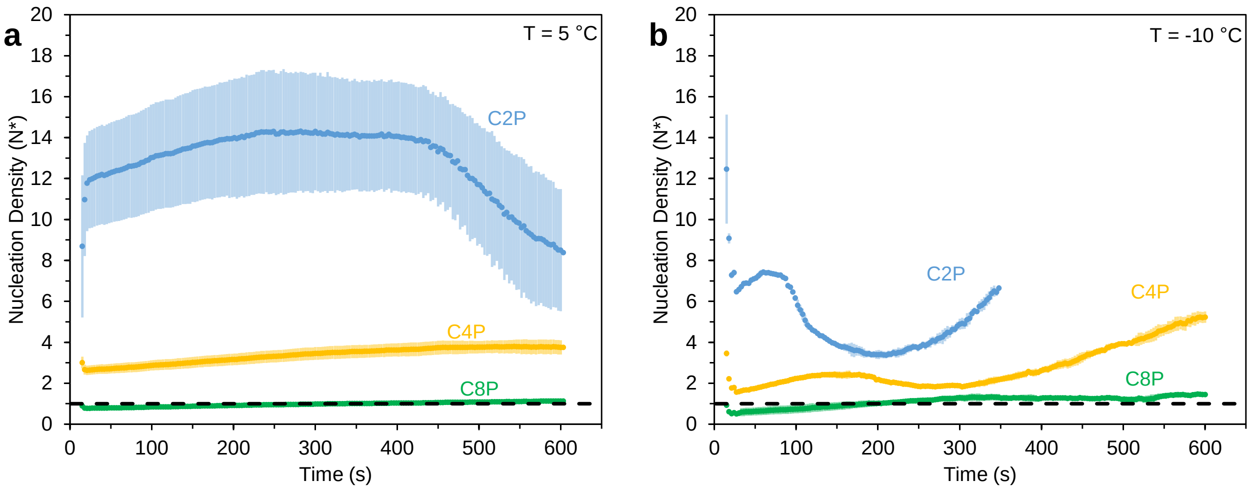

Supplementary Figure S7: The dimensionless nucleation density, where $N^*(t)$ is the ratio of droplets nucleated on each patterned surface to the number of droplets nucleated on the uniformly hydrophobic control surface. (**a**) At lower supersaturations ($S = 1.2$), the nucleation density is significantly higher on the C2P and C4P surfaces, especially for C2P where $N^* > 10$. Conversely, for patterns with a larger pitch between features (C8P), $N^* < 1$ for the first several minutes of condensation. (**b**) The nucleation density is still larger for C2P and C4P compared to the control surface (C) at higher supersaturations ($S = 3.5$), but the effect is less pronounced. This is because droplets tend to nucleate on the hydrophilic patterns regardless of the extent of supersaturation, whereas more droplets can nucleate on the control surface at the higher supersaturation.

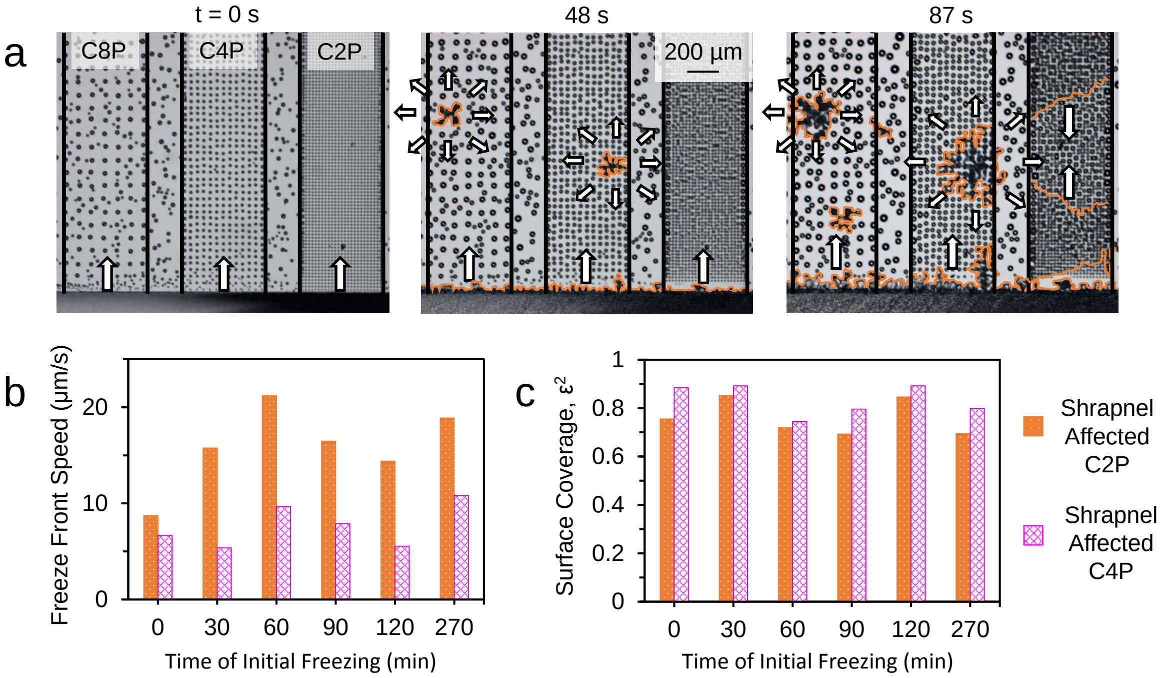

Supplementary Figure S8: Characterizing the "ice shrapnel" effect. (**a**) Under ambient conditions of $T_\infty = 21\,°\mathrm{C}$ and $H = 40\,\%$, the patterned substrate was cooled to a steady-state temperature of $T_s = -10\,°\mathrm{C}$. Immediately upon reaching steady-state, the thin film of supercooled water (bottom of screen) happened to freeze due to heterogeneous nucleation at the liquid-solid interface (1st frame). In the seconds after this initial freezing event, small pieces of ice appeared at random sites on the surface (second frame) which were likely ejected from the initial freezing event and are therefore termed "ice shrapnel." The frost proceeded to grow across the surface in two different ways: inter-droplet ice bridges that grew from the frozen film of water and additionally from the landing sites of the ice shrapnel (third frame). For clarity, the advancing frost fronts are outlined in orange. (**b,c**) To determine the dependence of pattern geometry and the time of freezing onset on the rate of frost growth, the thin film of supercooled water was intentionally frozen either 0, 30, 60, 90, 120, or 270 s after reaching a steady-state temperature of $T_s = -10\,°\mathrm{C}$. As expected, frost was able to grow more quickly across the C2P array of condensation compared to C4P due to the closer packing of the supercooled condensation. However, the rate of frost growth did not exhibit any clear dependence upon the time of condensation growth prior to freezing, due to the highly random interference of the ice shrapnel. See Supplementary Movie 8.

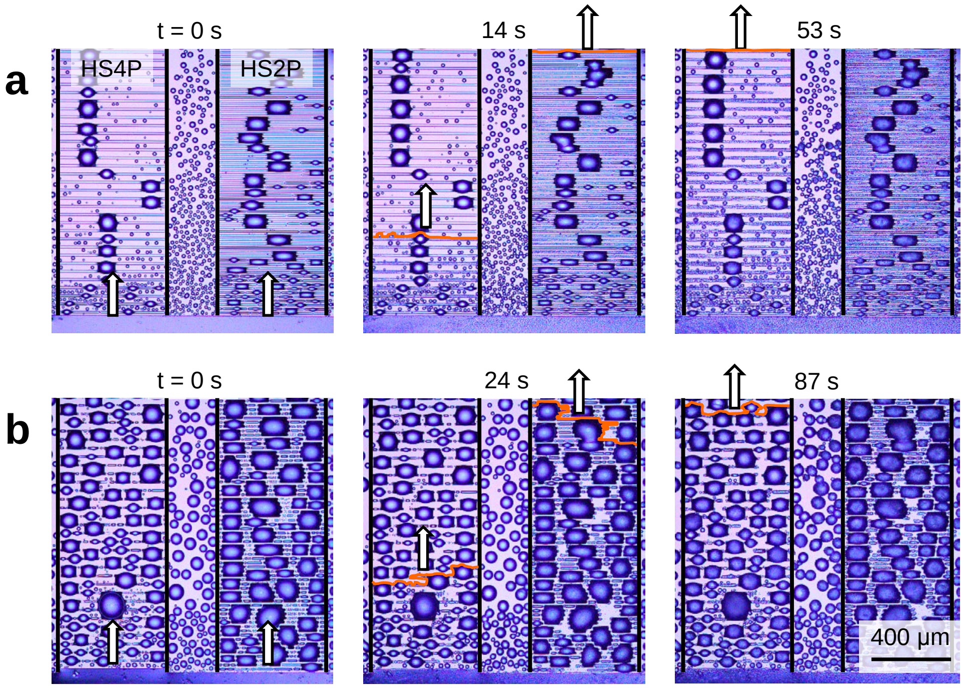

Supplementary Figure S9: Growth of inter-droplet frost across horizontal stripes of water (HS4P on left and HS2P on right). The ambient conditions were $T_\infty = 24.0\,^\circ\mathrm{C}$ and $H = 26\,\%$, such that the ice shrapnel effect was suppressed and all frost growth originated from the frozen pad of water (bottom of screen). Freezing was initiated either (**a**) 1 min or (**b**) 5 min after reaching a steady-state temperature of $T_s = -10\,^\circ\mathrm{C}$ by touching ice to the film of water bordering the stripe arrays. Unlike with the circle or triangle arrays (cf. Fig. 7), the average rate of frost growth did not increase at later freezing times. This is attributed to the growing bulges of water partially drying out the water along the stripes with increasing growth time. In other words, the increased ease of inter-droplet ice bridging along the larger bulges of water is cancelled out by an inhibited growth of ice across the stripes of water. See Supplementary Movies 11 and 12.

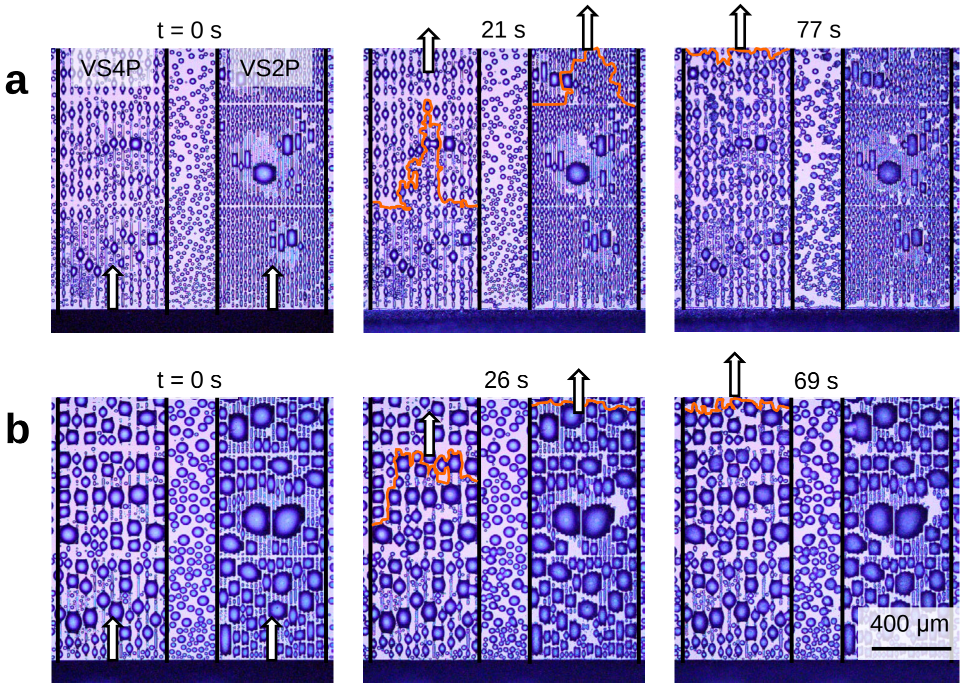

Supplementary Figure S 10: Frost growth across vertical stripes of water (VS4P on left and VS2P on right) under the same experimental conditions as detailed in Supplementary Fig. S9. Note that the vertical stripes of water were somewhat discontinuous, potentially due to minor surface contamination. These discontinuities prevent the ice from directly propagating across the lines; instead, inter-droplet ice bridges had to connect across the gaps. Similar to what was observed with the horizontal stripes, the rate of frost growth was not significantly different for freezing initiated at (**a**) 1 min compared to (**b**) 5 min. See Supplementary Movies 13 and 14.

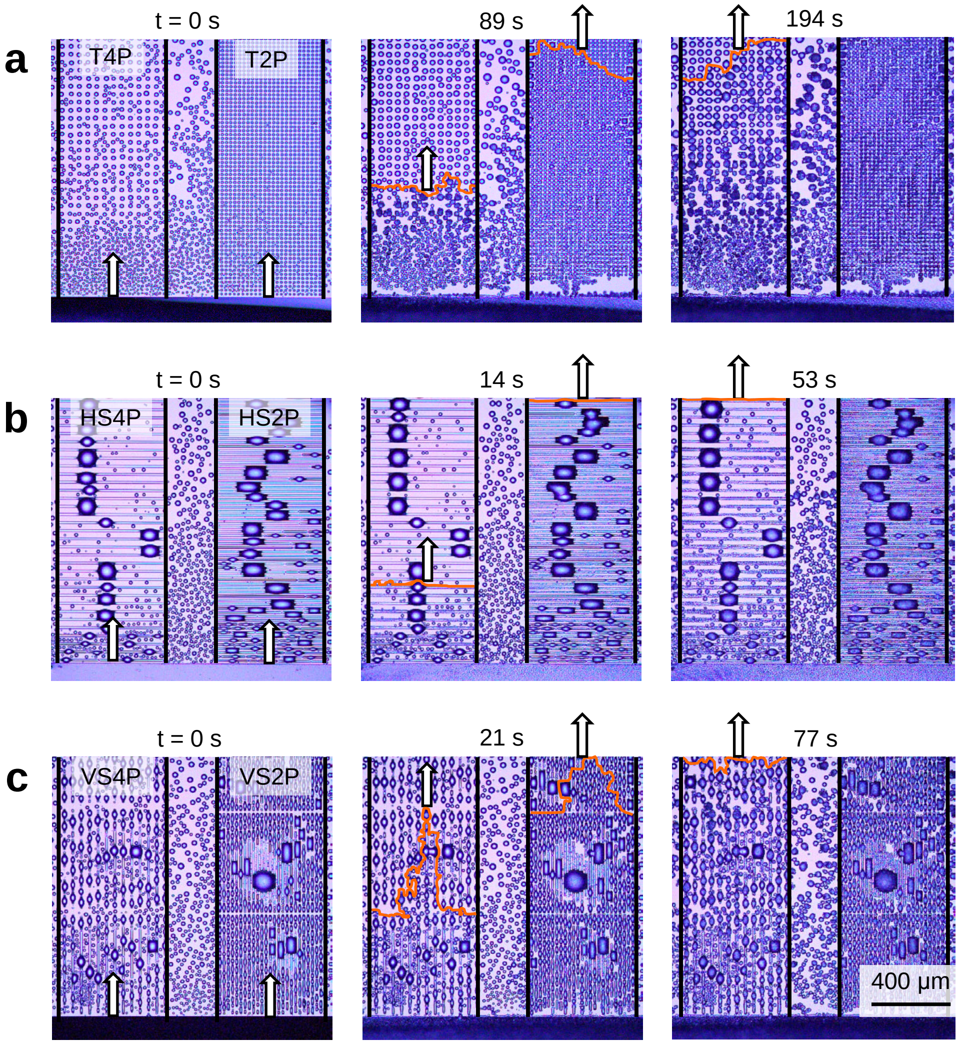

Supplementary Figure S11: A direct visual comparison of inter-droplet frost growth across (**a**) triangle arrays, (**b**) horizontal stripes, and (**c**) vertical stripes. Note that these figures were taken from Fig. 6a, Supplementary Fig. 9a, and Supplementary Fig. S10a, respectively. When the time of initial freezing is 1 min after reaching $T_s = -10\,^{\circ}\mathrm{C}$, the rate of frost growth was higher across the stripes by a factor of 3 compared to the discontinuous triangles. This is attributed to two primary factors: fewer inter-droplet ice bridges are required for the continuous stripe patterns, and the stripes exhibit a plateau surface coverage of water much faster than the triangles (cf. Figs. 2, 3). See Supplementary Movies 9, 11, and 13.

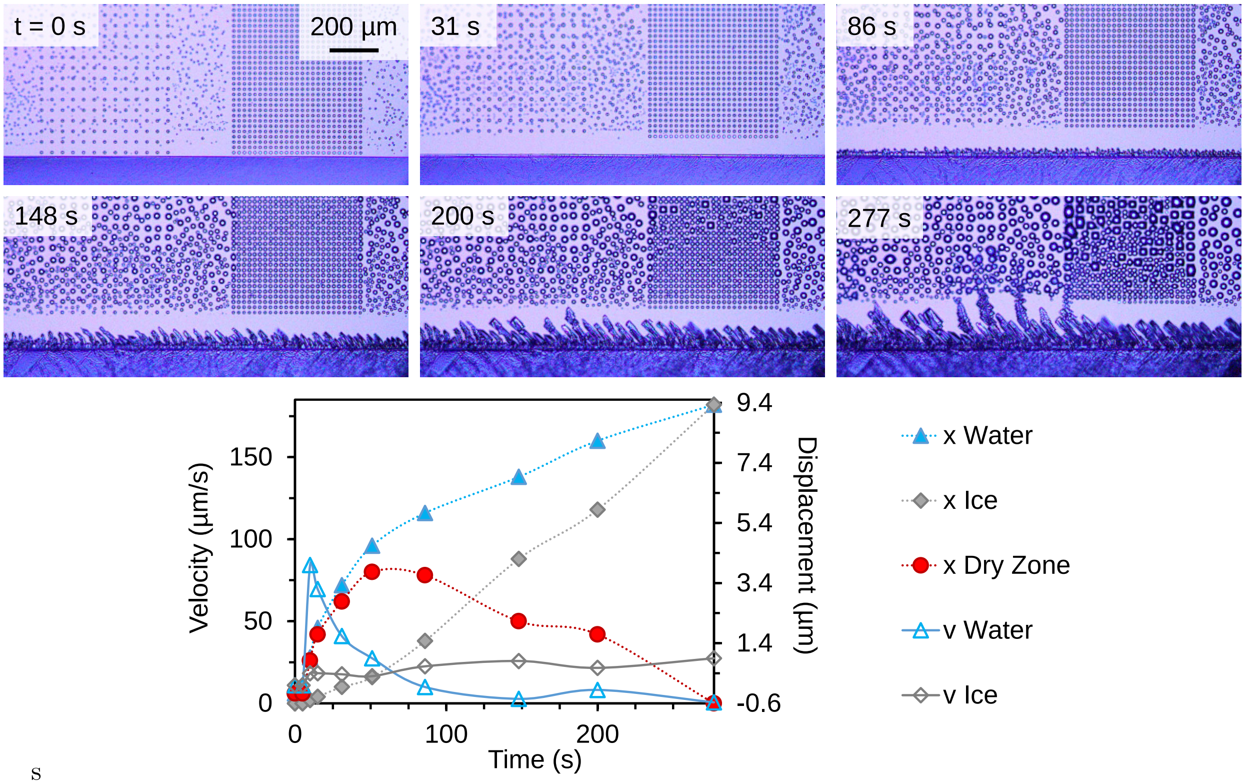

s

Supplementary Figure S12: Formation of a dry zone between a film of frozen water and arrays of supercooled condensate on triangular patterns. The film of water (bottom of screen) was frozen immediately after reaching $T_s = -10\,°\text{C}$ (1st frame) under ambient conditions of $T_\infty = 24.0\,°\text{C}$ and $H = 26\,\%$. Initially, the first eight rows of patterned condensate completely evaporated due to the vapor pressure gradient between the ice and water. However, eventually the growing frost was able to bridge all the way across the dry zone, resulting in the propagation of frost across the surface (last frame). Note that the shape of the advancing frost front has a finger-like geometry, due to the discontinuous pattern of liquid condensate being harvested. The positions and velocities of the receding water (triangles) and advancing frost (diamonds) are plotted as a function of time, where each data point corresponds to the complete evaporation of the next row of patterned condensate. The final data point corresponds to when the dry zone (circles) has completely collapsed due to ice bridging. See Supplementary Movie 16.

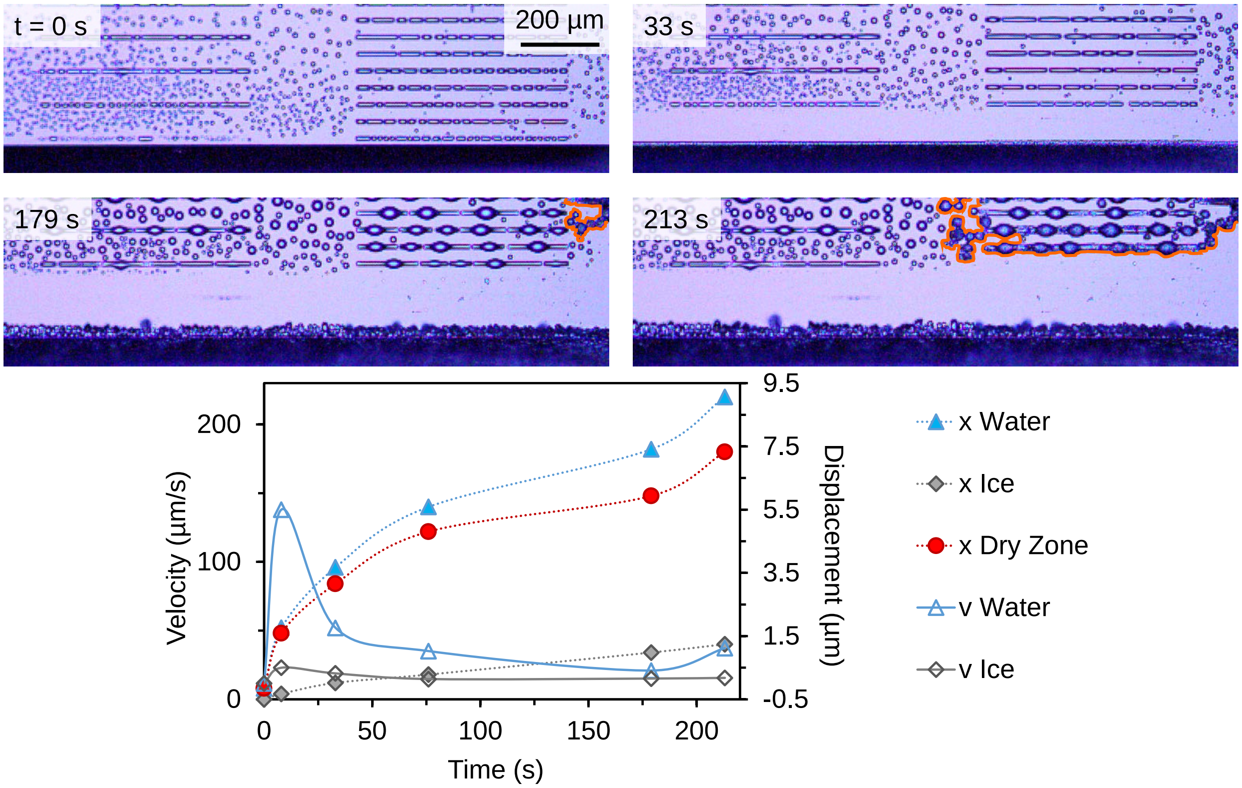

Supplementary Figure S 13: Formation of a dry zone between ice and horizontal stripes of supercooled condensate. Except for the different geometry of the patterned water, the experimental conditions and protocol are identical to those described in Supplemental Fig. 12. Note that the frost advancing into the dry zone is extremely uniform in its profile, owing to the stripes of harvested water running parallel to the ice. Each data point in the graph represents the next stripe of water having evaporated completely due to the water harvesting of the ice. The dry zone was extremely stable for several minutes; in fact, the remaining stripes of supercooled water only froze due to the invasion of frost from elsewhere on the surface (final frame, frost invasion outlined in orange). See Supplementary Movie 17.

Supplementary Movies:

Supplementary Movie 1:
Growth of condensation on C2P (left), C4P (middle), and C8P (right) chemical patterns at T_s = 5 °C (S = 1.2). The video was captured at 0.33 fps and played back at 20 fps (60X fast forward). The field of view is 2.46 × 1.84 mm.

Supplementary Movie 2:
Growth of condensation on C2P (left), C4P (middle), and C8P (right) chemical patterns at T_s = −10 °C (S = 3.5). The video was captured at 0.33 fps and played back at 20 fps (60X fast forward). The field of view is 2.46 × 1.84 mm.

Supplementary Movie 3:
Growth of condensation on HS2P (left), HS4P (middle), and HS8P (right) chemical patterns at T_s = 5 °C (S = 1.2). The video was captured at 0.33 fps and played back at 20 fps (60X fast forward). The field of view is 2.46 × 1.84 mm.

Supplementary Movie 4:
Growth of condensation on HS2P (left), HS4P (middle), and HS8P (right) chemical patterns at T_s = −10 °C (S = 3.5). The video was captured at 0.33 fps and played back at 20 fps (60X fast forward). The field of view is 2.46 × 1.84 mm.

Supplementary Movie 5:
Growth of condensation on T2P (left), T4P (middle), and T8P (right) chemical patterns at T_s = 5 °C (S = 1.2). The video was captured at 0.33 fps and played back at 20 fps (60X fast forward). The field of view is 2.46 × 1.84 mm.

Supplementary Movie 6:
Growth of condensation on T2P (left), T4P (middle), and T8P (right) chemical patterns at T_s = −10 °C (S = 3.5). The video was captured at 0.33 fps and played back at 20 fps (60X fast forward). The field of view is 2.46 × 1.84 mm.

Supplementary Movie 7:
Growth of ice shrapnel on T8P (left), T4P (middle), and T2P (right) chemical patterns at T_s = −10 °C (S = 3.5). First, the film of supercooled water (bottom of the screen) froze due to heterogeneous nucleation. In the proceeding seconds, small pieces of ice appeared all over the surface, which is most likely ice shrapnel that was ejected from the explosive freezing of the water film. This ice shrapnel was able to grow across the surface due to inter-droplet ice bridging. The portion of the video that corresponds to the cropped images in Fig. 5 is located at the upper-left portion of the screen. The video was captured at 0.2 fps and played back at 20 fps (100X fast forward). The field of view is 2.46 × 1.84 mm.

Supplementary Movie 8:
Growth of ice shrapnel on C8P (left), C4P (middle), and C2P (right) chemical patterns at T_s = −10 °C (S = 3.5). Freezing was triggered at the film of supercooled water (bottom

of the screen) as soon as the surface reached the steady-state temperature. As with Supplementary Movie 7, ice shrapnel appeared in multiple sites on the surface after the initial freezing event, resulting in multiple waves of inter-droplet frost growth propagating across the surface. The video was captured at 0.33 fps and played back at 20 fps (60X fast forward). The field of view is 2.46 × 1.84 mm.

Supplementary Movie 9:
Propagation of frost across supercooled condensate on T4P (left) and T2P (right) chemical patterns at $T_s = -10$ °C (S = 2.7). The onset of freezing was triggered at the thin film of water (bottom of screen) 1 min after reaching steady-state temperature. The video was captured at 1 fps and played back at 30 fps (30X fast forward). The field of view is 2.05 × 1.54 mm.

Supplementary Movie 10:
Propagation of frost across supercooled condensate on T4P (left) and T2P (right) chemical patterns at $T_s = -10$ °C (S = 2.7). The onset of freezing was triggered at the thin film of water (bottom of screen) 5 min after reaching steady-state temperature. The video was captured at 1 fps and played back at 30 fps (30X fast forward). The field of view is 2.05 × 1.54 mm.

Supplementary Movie 11:
Propagation of frost across supercooled condensate on HS4P (left) and HS2P (right) chemical patterns at $T_s = -10$ °C (S = 2.7). The onset of freezing was triggered at the thin film of water (bottom of screen) 1 min after reaching steady-state temperature. The video was captured at 1 fps and played back at 30 fps (30X fast forward). The field of view is 2.05 × 1.54 mm.

Supplementary Movie 12:
Propagation of frost across supercooled condensate on HS4P (left) and HS2P (right) chemical patterns at $T_s = -10$ °C (S = 2.7). The onset of freezing was triggered at the thin film of water (bottom of screen) 5 min after reaching steady-state temperature. The video was captured at 1 fps and played back at 30 fps (30X fast forward). The field of view is 2.05 × 1.54 mm.

Supplementary Movie 13:
Propagation of frost across supercooled condensate on VS4P (left) and VS2P (right) chemical patterns at $T_s = -10$ °C (S = 2.7). The onset of freezing was triggered at the thin film of water (bottom of screen) 1 min after reaching steady-state temperature. The video was captured at 1 fps and played back at 30 fps (30X fast forward). The field of view is 2.05 × 1.54 mm.

Supplementary Movie 14:
Propagation of frost across supercooled condensate on VS4P (left) and VS2P (right) chemical patterns at $T_s = -10$ °C (S = 2.7). The onset of freezing was triggered at the thin film of water (bottom of screen) 5 min after reaching steady-state temperature. The

video was captured at 1 fps and played back at 30 fps (30X fast forward). The field of view is 2.05 × 1.54 mm.

Supplementary Movie 15:
Formation of a dry zone between ice (bottom of screen) and condensate on VS4P (left) and VS2P (right) chemical patterns at $T_s = -12.5$ °C. The dry zone occurred because freezing was triggered in the water film (bottom of screen) as soon as the surface reached $T_s = -5$ °C, such that the size of condensate on the patterns was minimal. After freezing onset, the water harvested by the ice was not sufficient to bridge across the gap to the stripes, resulting in a dry zone that was stable for several minutes. Frost eventually invaded the stripes of water due to ice bridges propagating from outside of the field-of-view. The video was captured at 1 fps and played back at 30 fps (30X fast forward). The field of view is 2.05 × 1.54 mm.

Supplementary Movie 16:
Formation of a dry zone between ice (bottom of screen) and condensate on C4P (left) and C2P (right) chemical patterns at $T_s = -10$ °C (S = 2.7). The initial freezing of the water film (bottom of screen) was triggered as soon as the surface reached $T_s = -10$ °C. The water harvested by the ice initially resulted in a dry zone, but the growing finger formations of the frost were able to bridge across the gap and connect to the water patterns. The video was captured at 1 fps and played back at 30 fps (30X fast forward). The field of view is 2.05 × 1.54 mm.

Supplementary Movie 17:
Formation of a dry zone between ice (bottom of screen) and condensate on HS8P (left) and HS4P (right) chemical patterns at $T_s = -10$ °C (S = 2.7). The initial freezing of the water film (bottom of screen) was triggered as soon as the surface reached $T_s = -10$ °C. The water harvested by the ice resulted in a dry zone that was stable for several minutes. The shape of the growing frost was much more uniform compared to the Supplemental Movie 16, due to the water harvesting coming from stripes of water running parallel to the ice. Frost eventually invaded the stripes of water due to ice bridges propagating from outside of the field-of-view. The video was captured at 1 fps and played back at 30 fps (30X fast forward). The field of view is 2.05 × 1.54 mm.
